# The bacterial genetic determinants of *Escherichia coli* capacity to cause bloodstream infections in humans

**DOI:** 10.1371/journal.pgen.1010842

**Published:** 2023-08-02

**Authors:** Judit Burgaya, Julie Marin, Guilhem Royer, Bénédicte Condamine, Benoit Gachet, Olivier Clermont, Françoise Jaureguy, Charles Burdet, Agnès Lefort, Victoire de Lastours, Erick Denamur, Marco Galardini, François Blanquart, Colibafi/Septicoli & Coliville groups

**Affiliations:** 1 Institute for Molecular Bacteriology, TWINCORE Centre for Experimental and Clinical Infection Research, a joint venture between the Hannover Medical School (MHH) and the Helmholtz Centre for Infection Research (HZI), Hannover, Germany; 2 Cluster of Excellence RESIST (EXC 2155), Hannover Medical School (MHH), Hannover, Germany; 3 Université Sorbonne Paris Nord, INSERM, IAME, Bobigny, France; 4 Université Paris Cité, INSERM, IAME, Paris, France; 5 Département de Prévention, Diagnostic et Traitement des Infections, Hôpital Henri Mondor, Créteil, France; 6 Unité Ecologie et Evolution de la Résistance aux Antibiotiques, Institut Pasteur, UMR CNRS 6047, Université Paris-Cité, Paris, France; 7 Laboratoire de Génétique Moléculaire, Hôpital Bichat, AP-HP, Paris, France; 8 Center for Interdisciplinary Research in Biology, Collège de France, CNRS UMR7241 / INSERM U1050, PSL Research University, Paris, France; University of Warwick, UNITED KINGDOM

## Abstract

*Escherichia coli* is both a highly prevalent commensal and a major opportunistic pathogen causing bloodstream infections (BSI). A systematic analysis characterizing the genomic determinants of extra-intestinal pathogenic vs. commensal isolates in human populations, which could inform mechanisms of pathogenesis, diagnostic, prevention and treatment is still lacking. We used a collection of 912 BSI and 370 commensal *E*. *coli* isolates collected in France over a 17-year period (2000–2017). We compared their pangenomes, genetic backgrounds (phylogroups, STs, O groups), presence of virulence-associated genes (VAGs) and antimicrobial resistance genes, finding significant differences in all comparisons between commensal and BSI isolates. A machine learning linear model trained on all the genetic variants derived from the pangenome and controlling for population structure reveals similar differences in VAGs, discovers new variants associated with pathogenicity (capacity to cause BSI), and accurately classifies BSI vs. commensal strains. Pathogenicity is a highly heritable trait, with up to 69% of the variance explained by bacterial genetic variants. Lastly, complementing our commensal collection with an older collection from 1980, we predict that pathogenicity continuously increased through 1980, 2000, to 2010. Together our findings imply that *E*. *coli* exhibit substantial genetic variation contributing to the transition between commensalism and pathogenicity and that this species evolved towards higher pathogenicity.

## Author summary

*Escherichia coli* is a usually harmless bacteria typically found in the human gut. However, certain strains can cause severe bloodstream infections (BSIs) with high mortality rates, especially in older and fragile patients. To develop more effective prevention and treatment measures, understanding the genetic factors that determine a strain’s capacity to cause infection is essential.

In this study, we analyzed 912 BSI and 370 commensal *E*. *coli* isolates collected in France over 17 years (2000–2017). Through genetic comparison, we identified notable differences in their genetic backgrounds, as well as the presence of virulence-associated genes (VAGs) and antimicrobial resistance (AMR) genes. Using a machine learning model, we distinguished BSI from commensal strains, thereby discovering genetic factors linked to pathogenicity.

Our findings indicate that *E*. *coli* exhibits significant genetic variation contributing to the switch between commensalism and pathogenesis. We also suggest that pathogenicity in France has steadily increased over time, presenting a potentially serious public health threat. Our work is an important step in guiding future research to enhance diagnostic, prevention, and treatment strategies.

## Introduction

*Escherichia coli* bloodstream infections (BSI) are severe diseases with an incidence of around 5 × 10^−4^ to 1 × 10^−3^ per person-year in Europe and the United States and a mortality ranging from 10 to 30% [1–5]. They may account for a few percents of all deaths in these countries [4]. The increase in incidence of BSI [1,2], the global emergence of multidrug resistance clones such as ST131 [6–9], and the aging population all make BSI an important and growing public health problem. A better understanding of the bacterial genetic factors determining pathogenicity (the capacity to cause infection) and virulence (the severity of infection) [10] would improve our understanding of pathophysiology and potentially improve stewardship and control policies.

The primary niche of *E*. *coli* is the gut of vertebrates, especially humans, where it behaves as a commensal [11]. BSI are opportunistic infections resulting from two main routes of infection, digestive and urinary, corresponding to two distinct pathophysiologic entities. BSI with digestive portal of entry are more severe than urinary ones: for example, the respective death rates were 14.7 vs 7.6% in a study in France [12]. Host conditions and comorbidities affect the severity of infection [13–15]. A few bacterial genetic factors affecting virulence have been reported. In a genome-wide association study (GWAS) conducted on 912 patients, no bacterial genetic factor was associated with outcome (death, septic shock, admission to ICU), possibly because of insufficient power [16]. Alternatively, in a murine model of BSI, a GWAS conducted on 370 *Escherichia* strains have shown that the *Yersinia pestis* High Pathogenicity Island (HPI), and two additional groups of genes involved in iron uptake, were associated with a higher probability of mouse death [17].

There is a rich tradition of comparing *E*. *coli* strains sampled from commensal carriage vs. in infections to reveal the determinants of pathogenicity [18,19], classically defined as the propensity to cause infection [10]. The numerous previous studies investigating the bacterial genetic determinants of pathogenicity vary in their study design, and in the resolution of the bacterial genetic information. Many studies use a case-control design, where cases are the individuals with BSI, controls are the healthy individuals, and the exposure is *E*. *coli* sampled from the blood or stool, respectively [20–25]. The exposure is variable, because bacteria are genetically variable. In this design, it is important to adjust for any potential confounder. Indeed, host factors, such as age or co-morbidities, are important determinants of infection [12]. Without adjustment, it is possible that the bacterial genetic factors identified do not causally affect pathogenicity but rather are associated with colonization of at-risk host groups. A less frequent design consists in sampling *E*. *coli* from stools vs. from infections in the same individuals, similar to a case-crossover design [18,26–29]. This design is interesting because it removes the confounding effect of the host factors. The case-crossover design, however, has limited power to detect variants associated with infections because it only considers hosts with infections, limiting the possibility of comparison to the diversity of strains present in stools of these hosts. For example, if hosts are colonized by a single strain which is the source of infection, the case-crossover design has zero power to detect genetic variants affecting pathogenicity as the strains from stool and blood samples will be identical.

Previous studies also differ in the genetic characterization of *E*. *coli*. Many studies characterize known virulence or resistance genes and alleles, serotypes, phylogroups or sequence types. This prevents the discovery of new determinants of pathogenicity beyond the established lists of virulence and resistance genes. The limited genetic information also prevents controlling for bacterial population structure. Indeed, the increased availability of large whole genome sequence collections from BSI revealed that a small number of sequence types, mainly ST131, 73, 95, 69, 10, are involved in the majority of BSI [30]. These STs are rich in virulence associated genes (VAGs) encoding adhesins, iron acquisition systems, protectins and toxins [18,19]. Pinpointing potentially causal individual genetic determinants can only be done in a rigorous GWAS controlling for population structure. Such control would also estimate the heritability, which is the fraction of variance in pathogenicity explained by bacterial genetic factors.

Thus, no study has so far investigated the bacterial genetic determinants of pathogenicity by comparing large numbers of whole genome sequences of bacteria sampled from the gut (commensals) vs. sampled from infections. A large-scale, comprehensive and systematic picture of the bacterial genetic determinants of *E*. *coli* pathogenicity is missing. In the present work, we took advantage of two recently published collections of BSI [12,31] and commensal [32] strains gathered between 1980 and 2017 in France, with associated host metadata, and full genome sequences. We compared BSI and commensal strain genomes at three levels: phylogenomic composition, virulence and resistance gene content, and lastly unitig content in a GWAS. Our goal was to compare the diversity of commensal and BSI strains and to identify specific genomic features affecting the propensity to cause BSI, using both a targeted and a hypothesis-free approach.

## Results

### A dataset of 912 BSI and 370 commensal isolates

**Fig 1:**
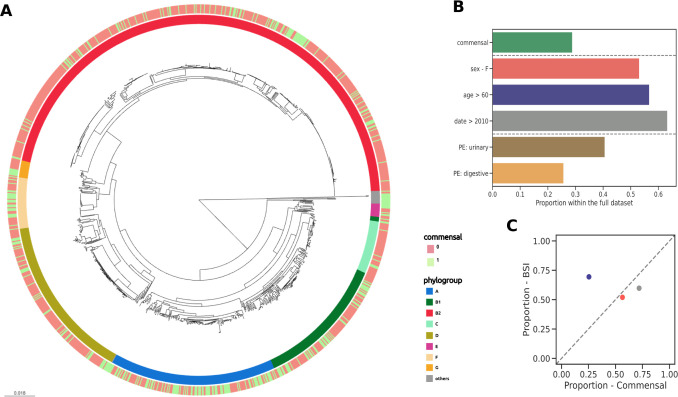
Global representation of the strain data set and the associated variables. (A) Core genome phylogenetic tree of the 1,282 *E*. *coli* isolates used in this study (see Materials and methods) with their phylogroup distribution (internal color ring) and commensal or BSI status (external color ring). (B) Proportion of commensal isolates, distribution of covariates (sex, age, collection date), and BSI isolates with the urinary tract and digestive tract as portal of entry within the full dataset. (C) Scatter plot of the distributions of all covariates in the two collections, colors matching that of panel B. PE: portal of entry.

We compared the genomes of 912 strains from BSI in adults, originating from two prospective multicentric studies (Colibafi in 2005 and Septicoli in 2016–7 [12,31]) performed in the Paris area, to the genomes of 370 commensal strains gathered from stools of healthy adult subjects in 2000, 2001, 2002, 2010 and 2017 in Brittany and the Paris area (**Fig 1A**). In-hospital death (or at day 28) was 12.9 and 9.5% in the Colibafi and Septicoli studies, respectively. Most of the BSI were community acquired (79.6 and 54.3% in the two collections, respectively). To avoid biases, all strains were isolated with similar protocols adapted to the sample origin (BSI and commensal) and sequenced in our laboratory using a similar approach (Illumina technology). To reduce the influence of the origin of the different studies we introduced the date of the study as a covariate, encoding it as a binary variable with the studies collected before and in or after 2010. To account for host factors, we additionally included sex and age as binary variables. For age, the variable was recording if the individual was above 60 years old or not. Finally, we also focused on the reported portal of entry of the BSI strains, which has previously been associated with some genetic variants (**Fig 1B**) [16]. The two collections had similar distributions of these variables, with the important exception of the proportion of isolates corresponding to older individuals, which is higher (69.43%) in the BSI collection (**Fig 1C**).

We computed the power of our design to detect variants affecting pathogenicity with simulations. Our design achieves a 50 to 60% power to detect a variant increasing pathogenicity by +30%. In our design, unadjusted confounders would unwantedly increase power and inflate the effect size estimate (**S1 Fig**). To inform potential future studies, we also compared our case-control study design to the other commonly used case-crossover design. In the case-crossover design, the commensal strains would have been hypothetically isolated from the 912 stool samples of the same BSI patients (instead of 370 unrelated healthy individuals). The case-crossover design removes the undesirable effects of confounding (**S1 Fig**). However, it suffers from low power when the within-host diversity of *E*. *coli* takes on plausible values of 1–5 strains per host, and underestimates effect size.

### Commensal strains are genetically more diverse than BSI strains and have a distinct phylogenetic composition

**Fig 2:**
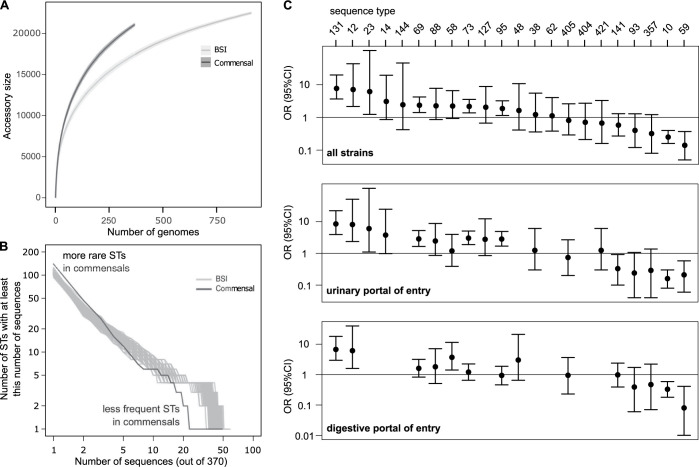
Comparison of the global phylogenomic characteristics of the commensal and BSI collections. (A) Pangenome sizes as a function of the number of genomes analyzed for the BSI (912 strains) and commensal (370 strains) collections, showing the greater pangenome size of the commensal collection. (B) Cumulative distribution of strain sequences within ST in commensal and BSI collections. To be able to compare the BSI collection with the smaller commensal collection (N = 370), we extracted 200 random sub-samples of 370 sequences from the BSI collection (grey curves). (C) Comparison of the distribution of the sequence types (STs) of the *E*. *coli* commensal and BSI collections isolates (see **S2 Table**). We show the odds ratio (OR with 95% CI) for the risk of infection associated with colonization by each ST (logistic model of infection status as a function of the ST). We selected the STs present in at least 5 strains in at least one of the two collections. STs are ordered by decreasing associated odds ratio for all strains.

We first compared the global phylogenomic characteristics of the two collections. The pangenomes of the BSI (N = 912) and commensal (N = 370) collections were composed of 24,321 and 22,373 genes, respectively. For a comparable number of strains, commensal strains had a higher diversity in gene content than BSI strains (**Fig 2A**). Conversely, the core genomes of both collections were similar (3,133 and 2,985 genes, respectively), and close to the core genome of *E*. *coli* species as a whole. In terms of SNP diversity of the core genome, the commensal collection was more diverse (pairwise nucleotide diversity π = 2.10e^-2^) than the BSI collection (π = 2.05e^-2^, p-value << 10e^-10^).

Commensal strains belonged almost equally to A and B2 phylogroups (25.4% and 32.4%) whereas BSI strains belonged mainly to phylogroup B2 and D (51.2% and 15.8%) (**Fig 1A** and **S1 Table**). The commensal collection was more diverse in its ST composition, with a higher number of rare STs and a lower number of frequent STs compared to the BSI collection (**Fig 2B**). This greater phylogenetic diversity could explain both the larger diversity in gene content [33] and larger nucleotidic sequence diversity of the pangenomes of commensals.

As previously noted, the diversity of STs in commensal strains was very distinct to that in BSI strains (**S2 Table**). Notably, ST10 and ST59 were abundant in commensal strains (13.2% and 3.8%) but under-represented in BSI strains (3.7% and 0.6%); on the contrary, ST131, ST73, ST69, ST95 were less common in commensal strains than they are in BSI strains. This comparison can be translated in an odds ratio for the risk of infection associated with gut colonization by each ST, which can be seen as a quantitative measure of pathogenicity. The sequence type ST131 was the most pathogenic and ST59 the least pathogenic (**Fig 2C** and **S2 Table**). When the portal of entry was considered for the ST distribution, a similar pattern was observed for both portals of entry as for the whole collection, although the significance level of the risk of infection might change (**Fig 2C** and **S2 Table**).

The distribution of the O-group diversity also differed between the commensal and the BSI collections (**S3 Table**). The four O-groups targeted by the recently developed bioconjugate vaccine ExPEC4V [34,35], O1, O2, O6 and O25 are the most abundant O-groups in the BSI collection. However, unlike the O-groups O6 and O25, the O-groups O1 and O2 are not particularly associated with BSI strains (**S3 Table**). In other words, these two O-groups are frequent in BSI because they are the two most frequent O-groups in commensalism, but are not particularly pathogenic.

### BSI strains are enriched in VAGs and antibiotic resistance genes (ARGs) as compared to commensal strains

**Fig 3:**
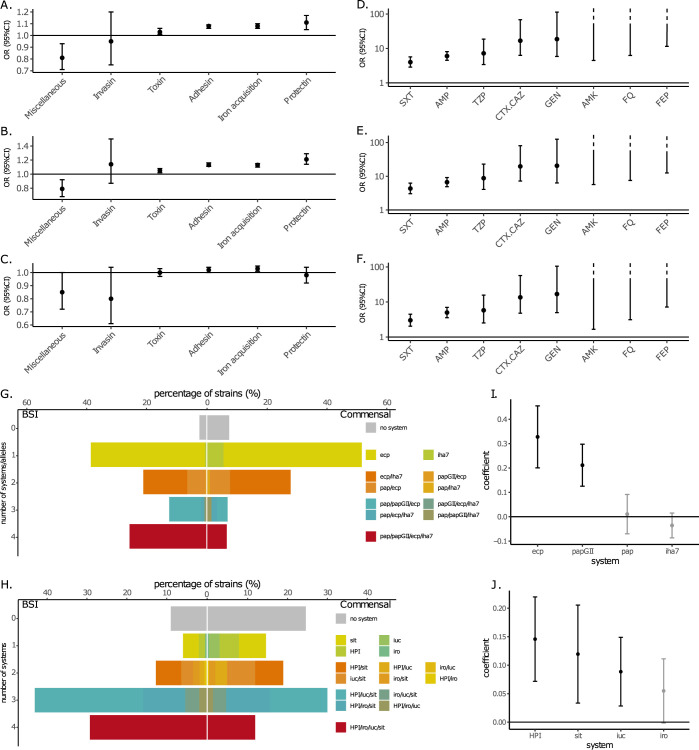
Comparison of known VAG and ARG characteristics studied in the targeted approach. (A-C) Comparison of the distribution of VAGs per strain among the six main functional classes of virulence of the *E*. *coli* commensal and BSI collections isolates. We show the odds ratio (OR with 95% CI) for the risk of infection associated with the number of VAGs (logistic model of infection status as a function of the number of VAGs), for (A) all the strains (912 BSI strains), (B) BSI strains with urinary portal of entry (PE) to commensals (498 BSI strains) and (C) BSI strains with digestive portal of entry to commensals (310 BSI strains). Functional classes of virulence are ordered by increasing associated odds ratio for all strains. (D-F) Comparison of the distribution of resistant strains for eight antibiotics of clinical importance of the *E*. *coli* commensal and BSI collections isolates. We show the odds ratio (OR with 95% CI) for the risk of infection associated with the resistance of strains (logistic model of infection status as a function of the resistance of strains), for (D) all the strains (912 BSI strains), (E) BSI strains with urinary portal of entry (PE) to commensals (498 BSI strains) and (F) BSI strains with digestive portal of entry to commensals (310 BSI strains). Categories of antibiotics are ordered by increasing associated odds ratio for all strains. For AMK, FQ and FEP categories, we only show the lower bound of the CI because the estimated odds ratios are huge as none of the commensal isolates were resistant to these antibiotics. AMK, amikacin; AMP, ampicillin; CTX/CAZ, cefotaxime/ceftazidime; FEP, cefepime; FQ, fluoroquinolones; GEN, gentamicin; SXT, cotrimoxazole; TZP, piperacillin/tazobactam. (G) Repertoire of adhesins and (H) iron capture systems in BSI and commensal strains. We selected the four most significant systems in terms of complete genes and/or alleles when comparing commensal to BSI strains (**S4 Table**) and evaluated their combinations. A system was considered present when all its genes were detected. (I) Predictor coefficients of pathogenicity among adhesins and (J) iron capture systems determined with a lasso regression. We calculated confidence intervals using 1000 bootstrap resamples. Unselected genes and/or alleles (lasso coefficient close to zero) are shown in gray.

Using a targeted approach, we next focused on the frequency of known VAGs and ARGs in both collections. A global comparison in the number of VAGs classified in functional categories showed a significantly higher presence of VAGs coding for adhesins, iron acquisition systems, protectins and toxins categories in BSI strains (**Fig 3A and S4 Table**). We found similar results when comparing against BSI strains with urinary portal of entry to commensals (**Fig 3B**). However, only the iron acquisition systems category remained significant when comparing against BSI strains with digestive portal of entry (**Fig 3C**). More precisely for the full dataset, the highest significance was observed for the *pap* genes with the *papGII* allele, followed by the *sit*, *iuc* and *irp2/fyuA* (HPI) genes, all with p-values << 10^−10^ (**S4 Table**). These analyses do not imply a causal role of these genes and alleles in BSI, as they are not adjusted for the distinct phylogenomic composition of commensal and BSI strains. However, it is possible to crudely adjust for this population structure by focusing on the B2 phylogroup strains which are known to exhibit the highest prevalence of VAGs within the *E*. *coli* species [19].

When only B2 phylogroup strains were compared, only VAGs coding for adhesins category remained significantly over-represented in BSI (**S2 Fig)**. When comparing only B2 strains with urinary portal of entry to B2 commensals, again only adhesins were over-represented, and no differences were observed when comparing only against B2 strains with digestive portal of entry (**S2 Fig**). Regarding individual genes, interestingly, for two VAGs with experimentally validated role in urinary tract infection, *pap* genes [36] and *fim* genes [37], we found a higher level of significance in B2 strains with urinary portal of entry than in all B2 strains (*pap*) or in all strains (*fimD-H*) (**S4 Table**).

As virulence in *E*. *coli* is the result of additive gene effect [38], we further evaluated the repertoire of adhesins and iron capture systems, the two categories for which we found the higher significance (**S4 Table**). We restricted our analysis to the four most significant systems in terms of complete genes and/or alleles (< 10^−7^). For both categories, we found a different distribution of systems between BSI and commensal strains, with more co-occurences of systems in BSI strains (**Fig 3** and **S5 Table**). For instance, 38% and 72% of BSI strains carry three or four systems of adhesins and iron capture systems respectively, compared to 13% and 42% respectively for commensal strains. We found two adhesins encoding genes, *ecp* and *papGII* and three iron capture systems, HPI and *sit* and *iuc* gene clusters to be the genes and/or alleles best explaining pathogenicity, using a lasso regression (with adhesins and iron capture systems evaluated separately or together) (**Fig 3I and 3J**). The *ecp* (or *yag* or *mat*) operon is highly prevalent within the *E*. *coli* species (more than 90%) and encodes a fimbrial adhesin (*E*. *coli* common pilus) used both by commensal and pathogenic strains [39,40]. In our work, the prevalence of *ecp* in commensal and BSI strains is 91 vs 98%, respectively, and the significance of its association with BSI strains disappeared when only the B2 were studied (**S5 Table**), suggesting a phylogenetic effect.

BSI strains were predicted to be more resistant to all classes of antibiotics than commensal strains (**Fig 3D**). This also holds true when specific portals of entry and/or phylogroup B2 were taken into account, with the exception of the resistance to amikacin when comparing B2 BSI strains with digestive portal of entry to B2 commensals (**Figs 3E, 3F and S3**). To verify that this over-representation of resistance in BSI was not explained by the fact that BSI isolates were slightly more recent on average than commensal isolates, we restricted our analysis to BSI Colibafi strains (sampled in 2005) and found the same results when considering all phylogroups and portals of entry, with the exception of amikacin and fluoroquinolones which had the lowest prevalence (**S3 Fig, panel D**).

No difference in VAG numbers (t-test, all Benjamini-Hochberg corrected p-value > 0.05), nor in resistance prevalences (Fisher’s exact test, all Benjamini-Hochberg corrected p value > 0.05), was found when comparing nosocomial and community BSI strains, considering both Septicoli (167 nosocomial and 296 community BSI strains) and Colibafi (75 nosocomial and 292 community BSI strains) collections together or individually.

### Bacterial genetic factors explain a large fraction of the variation in the BSI phenotype

**Fig 4:**
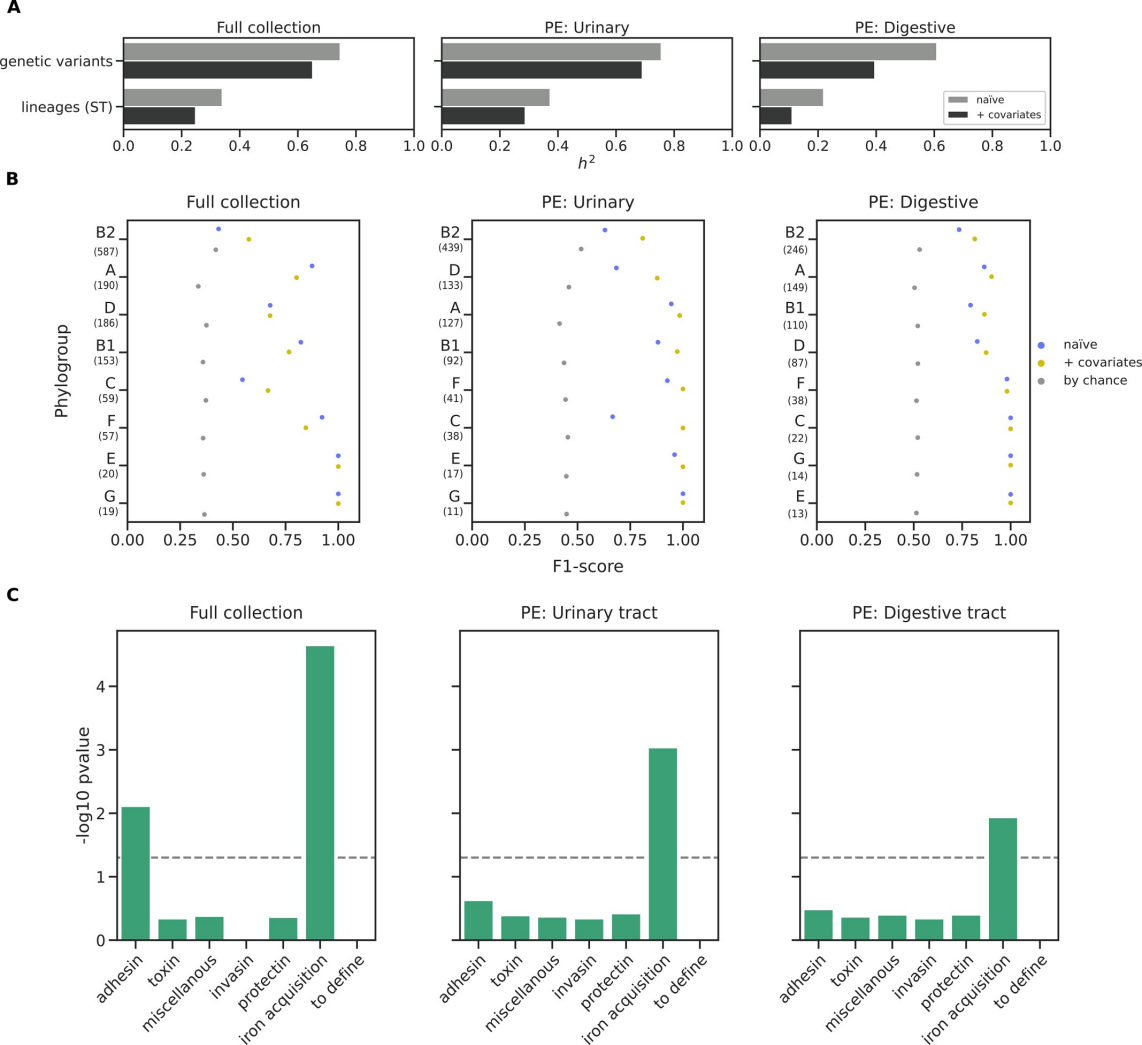
Main results of the hypothesis-free (GWAS) approach comparing commensal and BSI isolates using the portal of entry covariable. A) Heritability estimates for the commensal phenotype. B) wg-GWAS model performance within each phylogroup. F1-score representation for the naive analysis (blue dots), with covariates (yellow dots), and the one expected by chance (grey dots). Numbers within parentheses below each phylogroup indicate the sample size. C) Virulence associated genes enrichment analysis for the different functional categories. The significance threshold is represented over the dotted line (Fisher’s exact test, p<0.05). PE: portal of entry.

We then computed the heritability, as the proportion of the variance of a phenotype explained by variable genetic factors [41], to estimate whether we could expect to find bacterial genetic variants associated with commensalism vs. BSI in our dataset. We first measured the heritability using the ST information alone, to measure the influence of the genetic background on phenotypic variability. We then computed the heritability emerging from the individual genetic variants (**Fig 4A**). We found that STs could explain 24%, 28%, and 11% of the phenotypic variance in the full collection, the subset with BSI isolates with urinary tract as portal of entry and digestive tract as portal of entry, respectively. Genetic variants alone could explain a larger fraction of the phenotypic variability: 65%, 69%, and 39% in the full collection and the two subsets, respectively. This suggests that pathogenicity might not be solely determined by the sequence type but also by specific genetic variants within sequence types.

### A whole-genome machine learning model differentiates commensals from BSI strains

We applied a machine learning model trained on both the core and accessory genome of the strains to differentiate between commensal and BSI strains and highlight the genetic variants that contribute the most to the discriminatory power of the model (whole genome wg-GWAS). We performed the analysis on three different datasets: the full strain collection, and two subsets of BSI isolates: one with urinary tract as portal of entry, and another one with digestive tract as portal of entry. We used all the genetic variants covering the pangenome compactly represented by unitigs. Unitigs are nodes of sequence in a compressed de Bruijn graph, usually longer than k-mers, reducing the computational burden and the redundancy present in k-mer counting. They are short sequence fragments that represent both gene content and nucleotidic variation across genomes, including coding and intergenic non-coding sequences. We associated unitigs with phenotype with the elastic net linear model implemented in pyseer [42]. This approach is similar to a logistic regression except that the number of predictors (all genetic variants in the form of unitigs presence/absence) vastly exceeds the number of observations (phenotype, commensal vs. BSI). To resolve this problem, the likelihood of observations (the function to be maximized in classical logistic regression) is complemented by a term penalizing large coefficients: the ‘elastic net’ regularization. In pyseer, the strength of the penalty is tuned such as to maximize the accuracy of the fit when performing ‘leave-one-strain-out’ cross-validation. Fitting the elastic net model results in a set of unitigs retained in the whole genome model, with associated coefficients. The procedure ensures implicit correction for population structure and has been found to outperform other methods to detect causal variants [43,44].

We used the following three binary variables as covariates to account for host factors and collection biases: the sex of the individual, their age (older than 60 years old), and the date of each collection (before or after 2010). To quantify model performance, we computed the precision (proportion of true BSI among the predicted BSI strains), recall (sensitivity) and F1-score (harmonic mean of precision and recall) on each phylogroup (**Figs 4B and S4**).

The model performance improved in all cases when the covariates were considered for the associations, potentially confirming that host factors also explained part of bacterial pathogenicity. Model performance also improved in the two subsets with BSI isolates with a specific portal of entry, compared to the full collection. Furthermore, the model did not perform better than expected by chance within the B2 phylogroups and without dividing BSI by portal of entry (**Fig 4B**). This suggests the presence of specific genetic variants associated with either portal of entry, and underlines the critical importance of considering the portal of entry when inferring the determinants of pathogenicity [16].

We found a number of unitigs associated with commensalism vs. BSI (*i*.*e*. with non-zero weight in the elastic net model). Overall, 107 and 59 unitigs passed the threshold for the model built naïvely and with covariates, respectively, which we then mapped back to 34 and 28 genes. Moreover, we checked for gene hits upstream and downstream of the unitigs found in intergenic regions, revealing an additional set of 11 genes, one being also identified on coding region (*dhfr*). We found that 9 out of the 39 genes (28 in genes + 11 intergenic) obtained through the analysis with covariates were clearly related to virulence and/or resistance, notably the *iucB* gene encoding an aerobactin siderophore biosynthesis protein (30) and *papG* encoding the adhesin at the tip of the P pilus (31). Both the *iucB* and *papG* genes have already been associated with invasive uropathogenic *E*. *coli* (UPEC) isolates (15, 32). Of note, we had identified these two genes in the targeted approach even after focusing on the B2 phylogroup strains (see above). In addition to these two genes, we also found the following genes: *mltB*, which is part of a network connecting resistance, membrane homeostasis, biogenesis of pili and fitness in *Acinetobacter baumannii* [45]; *fliL*, encoding for the flagellar protein FliL [46]; *oprM*, as part of the intergenic hits, described as a component of an efflux pump in *Pseudomonas aeruginosa* [47] potentially involved in resistance to puromycin, acriflavine, and tetraphenylarsonium chloride (**S9 Table**). Lastly, two unnamed orthologous groups (group_5900 and group_9261), described as the putative bacterial toxin *ydaT* [48], were identified.

A larger number of genes were associated to the phenotype when dividing the BSI strains according to their portal of entry. We found a total of 152 and 96 associated unitigs for the urinary and digestive tract subsets, respectively, which we then mapped back to 101 and 45 genes, some of which are known to be involved in pathogenicity and antimicrobial resistance (**Table 1 and S5**). Additionally, 17 and 4 gene hits upstream and downstream of the unitigs in intergenic regions were found for the urinary and digestive tract subsets, respectively (**S9 Table**). Of note, the orthologous group 16391, identified for the urinary tract subset, was annotated as a paralog of *dhfR*, which had hits in its coding region. Moreover, we find *lysO* and *aqpZ* in both subsets.

**Table 1:** Genes with functions related to pathogenicity and antimicrobial resistance with unitigs associated with the phenotype mapped to them for the two subsets (urinary and digestive portal of entries).

Portal of entry: urinary tract		
**Gene**	**Relevance**	**Reference**
*papG*	Tip adhesin. Belongs to the *pap* operon encoding for a type P pilus	[16,52]
*papH*	Adhesin anchor to the cell. Belongs to the *pap* operon encoding for a type P pilus	[53]
*papF* *	Adhesin adapter. Belongs to the *pap* operon encoding for a type P pilus	[16,52]
*iucB/C*	Iron acquisition. Aerobactin siderophore biosynthesis protein	[54]
*mltC*	Involved in release of peptidoglycan-derived pathogen-associated molecular patterns as a virulence mechanism	[55]
*ompX*	Might be involved in biofilm formation and curli production	[56,57]
*dhfrI*, Group 16391 *	Trimethoprim resistance gene	[58], **S9 Table**
*fliD*	Relevance in adhesion. Flagellar hook-associated protein.	[59]
*dgcE*	Involved in regulation of the switch from flagellar motility to sessile behavior and curli expression	[60]
Groups 10969 and 4151	Type II/IV secretion system protein (T2SSE)	**S5 Table**
Group 9261	Putative bacterial toxin	**S5 Table**
*epsM*	Involved in type II secretion systems (T2SS)	**S5 Table**
*aceF*	Involved in the virulence and oxidative response of *P*. *aeruginosa*	[61]
*klcA*	Antirestriction protein. Encoding gene present in the *kilC* operon found in IncP plasmids, which usually carry multiple AMR determinants	[62]
**Portal of entry: digestive tract**		
**Gene**	**Relevance**	**Ref**
*iucC*	Iron acquisition. Aerobactin siderophore biosynthesis protein	[54]
Group 3130	Tfp pilus assembly protein FimV	**S5 Table**
*fliD*	Relevance in adhesion. Flagellar hook-associated protein	[59]
*epsE/F*	Type II/IV secretion system protein	**S5 Table**
*yehB*	Relevance in adhesion. Encodes a type of putative fimbrial complex belonging to the chaperone-usher assembly pathway	[63]
*oprM* *	Possibly involved in resistance to puromycin, acriflavine, and tetraphenylarsonium chloride. Component of an efflux pump in Pseudomonas aeruginosa.	**S9 Table** [47]

Intergenic (*) corresponds to gene hits upstream or downstream of associated unitigs within intergenic regions.

Taken as a whole, we found the associated genes to be enriched in the L COG category (replication, recombination and repair) for the three subsets, and in the K COG category (transcription) for the full dataset only. We also performed a Gene Ontology (GO) term enrichment analysis and found that for the subset with BSI isolates with urinary tract as portal of entry, the relevant (depth > 1) enriched GO terms include different categories related to metabolic processes, ion binding and intracellular anatomical structure (**S6 Table**). Similarly, to the targeted analysis described above, we found that the genes resulting from the three associations were enriched for VAGs and ARGs (**Fig 4C**); when considering all VAGs and ARGs together we found a significant (p-value < 0.05) enrichment for the full dataset and the urinary tract subset. We found VAGs related to iron acquisition to be enriched in all three datasets, while adhesins were enriched in the full dataset only. For the ARGs, only the resistance to cotrimoxazole (*dfrA* for SXT resistance) was enriched in the urinary tract subset.

The model can be used to predict the potential pathogenicity of other isolates based on the presence of the unitigs for which the model’s weight is different than zero. We predicted the pathogenicity of commensal strains collected at three time periods: 1980 [49], 2000–2002 and 2010. Interestingly, the model predicts a marked increase in pathogenicity of these commensal isolates, with the proportion doubling between the 1980s, the 2000s, and the 2010s (23% vs. 31% vs. 46%, **S5 Fig**). As the strains from the 1980 collection (VDG strains) have been stored in stabs between 1980–2000 before being frozen at -80°C, we verified that artifacts due to storage were not involved. We first confirmed the good quality of the strain sequences in all collections (**S6 Fig, panel A**). We then looked at the mutation patterns in the *rpoS* gene that are indicative of poor sample management [50] which may have affected genome content between sample isolation and sequencing (**S6 Fig, panel B**). As expected, we found a high rate of *rpoS* mutations in the VDG collection only. In addition, we compared the presence of two VAGs, *hlyC* (plasmid-borne or chromosomal) and *papC* (chromosomal), determined from this work by WGS and both phenotypically and via PCR in earlier studies [49,51]. We found a perfect match for PCR except one gene in one strain and slightly smaller percentages of presence of Hly and Pap assessed phenotypically (4.7% and 7%, respectively) versus 9% for both *hly* and *pap* assessed by WGS in this work. We speculated that different storage conditions could have caused the loss of virulence genes in the past collection, and biased downwards the predicted pathogenicity in 1980: although there was a weak (not significant) trend of increased gene content, this trend together with the inferred effects did not result in any change in predicted pathogenicity (Material and Methods). In sum, although the strains from 1980 were stored differently, there was no evidence that this could have affected our results on pathogenicity. Altogether, these data suggest that the commensal strains inhabiting the gut of healthy humans may have evolved towards higher pathogenicity in the past decades.

## Discussion

It is known since the 1940s [64] that within the *E*. *coli* species, some strains with a specific genetic background have higher capacity to cause extra-intestinal diseases. Later on, pathogenicity has been associated with specific serotypes, STs, and the phylogroup B2, which are enriched in some VAGs [65,66]. However, disentangling the respective roles of causal genetic variants from the genetic background in a mostly clonal species is a difficult task [67], and a comprehensive and systematic view of the bacterial genetic determinants of pathogenicity is lacking. To do so, we systematically investigated the genomic differences between 912 *E*. *coli* strains from bloodstream infections and 370 strains sampled from the stools of healthy volunteers. The large size of the collection and case-control study design ensure a powerful examination of these determinants.

We revealed differences at three levels. First, at the phylogenetic level, strains from BSI are less diverse, dominated by a small number of highly pathogenic STs, and consequently have smaller pangenomes and lower genetic diversity than commensal strains. Second, strains from infections are enriched in VAGs, and are predicted to be more antibiotic resistant. Third, in a machine learning assisted GWAS designed to identify putative causal variants, we found 118 and 49 genes associated with BSI with urinary and digestive portal of entry, respectively, independently of the clonal background. Our analyses give several new insights on *E*. *coli* pathogenesis: pathogenicity is a highly heritable phenotype, with 69% heritability for urinary tract portal of entry BSI; tens of specific variants may causally impact pathogenicity; antimicrobial resistance genes are associated with, but do not play a causal role in infection; and pathogenicity may have increased in the past decades in France.

We discuss these four new results in detail in the following. However, note that an important limitation of our study is that we did not use available information on host co-morbidities in BSI patients for the comparison with commensal strains. In fact, the most frequent co-morbidity in the BSI collection is immunosuppression, which was an exclusion criterion for the commensal collection. Co-morbidities are associated with BSI [5,12,31]. It is possible that co-morbidities act as a confounder in our study, if they both increase the probability of BSI and influence the *E*. *coli* strains carried by individuals. If this is the case, the variants we identify may not be directly causal for infections. Rather, they may be bacterial variants that favor the colonization of individuals with co-morbidities. Age is also associated with BSI [5,15]. In this work, we do control for age, albeit in a crude way, with the covariate “above or below 60 years old”. If some of the variation associated with age is not captured by this covariate, some of the variants we identify could favor the colonization of older or younger individuals. For example, there is evidence of age-associated variants in *Streptococcus pneumoniae* [68]. To attenuate these concerns on confounding, we remind that several of the significant variants have an experimentally validated role in infection and virulence (**Table 1**).

The heritability of pathogenicity is estimated at 69% (urinary PE) and 39% (digestive PE), in agreement with the higher role of the host factors in BSI with digestive PE [12,31]. Thus, a large fraction of pathogenicity is explained by bacterial genetic factors. This is roughly double of the heritability when considering STs alone, suggesting that specific genetic variants at a finer phylogenetic scale than ST are determining pathogenicity. For comparison, age, a host factor strongly associated with BSI, explains 17.6% of the variance. Bacterial genetics has a significant role in determining pathogenicity, even after basic host factors (age and sex) have been accounted for. The present study compares *E*. *coli* whole genomes in colonization and in infection in a case-control study, as done before for *Klebsellia pneumoniae* [69], *S*. *pneumoniae* [70], *Staphylococcus aureus* [71,72], *Enterococcus faecalis* [73], *Neisseria meningitidi*s [74]. These previous GWAS studies presented a range of results, from low heritability (2.6% for *S*. *aureus* carriage vs. BSI [71]), to intermediate (34% for *E*. *faecalis* intestinal colonization vs. extraintestinal infection, 36.5% for *N*. *meningitidis* carriage vs. invasive meningococcal disease), and an analogously large heritability of 70% for *S*. *pneumoniae* invasive disease vs. carriage, along with a handful of significant SNPs [70]. We find a large heritability for *E*. *coli* BSI vs. colonization, which suggests that a vaccine targeted at virulence determinants could reduce (at least temporarily) the burden of infection [34].

Some of the specific variants identified in the GWAS are involved in adhesion and in iron acquisition, as well as other functions. Generally, genes with a significant association are enriched in iron acquisition system, the L COG category (replication, recombination and repair) and GO terms including different categories related to metabolic processes, ion binding and intracellular anatomical structure.

Interestingly, 28% of identified genetic variants linked to pathogenicity between commensal and BSI isolates were located in intergenic regions. Non-translated intergenic regions compose 10–15% of bacterial genomes, and contain many regulatory elements with key functions. They have been shown to be under strong purifying selection in several bacterial species including *E*. *coli* [75]. This could indicate an important role of regulation of VAGs but also of core genome genes in pathogenicity [76]. Differences in gene (VAG, metabolic gene) regulation between anatomical sites have been reported in *Campylobacter coli* [77], *Klebsiella*, *Staphylococcus aureus* and *Streptococcus pyogenes* [78]. Also, differences between *E*. *coli* lineages have been described [40,79,80]. In the latter cases concerning differential expression of fimbriae (ECP, Ucl and P fimbriae, respectively), causal SNPs in the gene promoter region were identified. Further studies on the intergenic regions highlighted in the present analysis should be performed.

We found that strains from infections are more likely to be resistant to antimicrobials. What is the mechanism behind this association, also found in similar GWAS conducted on other pathogens [69,71]? Confounding is a first possibility: hosts with co-morbidities are more likely to develop a BSI and to use antibiotics frequently. Individuals may even be already treated by antibiotics at the time of infection, in which case only resistant strains would be able to cause this infection. If this mechanism operates, we could expect resistance to be more frequent in hospital-associated than in community-associated BSI, if hosts in hospitals are more likely to use antibiotics at the time of infection. However, we did not find any difference between resistance in hospital-associated and community-associated BSI. Second, antimicrobial resistance genes may have a causal role in infection. This seems unlikely given their very specific function. Third, there might be a genetic association (linkage disequilibrium) between resistance genes and genetic determinants of infection [69,81]. In the third case, we expect the association to disappear when controlling for population structure. With this control, we find that indeed, only one out of nine categories of resistance was significantly enriched in BSI compared to commensals. This suggests that antibiotic resistance genes are genetically linked with pathogenicity determinants, and opens the interesting possibility that antibiotic resistance coevolves with pathogenicity determinants associated with the clonal background of *E*. *coli*. The co-evolution of resistance and virulence elements may result in their co-localisation on the same genetic elements, such as plasmids, or nearby on the chromosome [82–84]. However, the properties of bacterial recombination enable associations to emerge even between physically distant genes [85]. We investigated the proximity between VAGs and AMR in our collections, and found that VAGs and AMR genes are never encoded in the same contig in the draft genomes used in this study, supporting the hypothesis of co-evolution of physically distant resistance and virulence elements through homologous recombination.

The large heritability of *E*. *coli* capacity to cause infection also implies that this trait can readily evolve. Evolution of *E*. *coli* pathogenicity would have important public health implications, given that *E*. *coli* BSI are a major cause of morbidity and death in Western countries. To investigate temporal trends in pathogenicity, we computed the pathogenicity score with the machine learning model (used to predict the commensal vs. BSI status of strains), in a dataset of commensals from 1980 to 2010 in France [32]. We found that the proportion of commensal *E*. *coli* isolates predicted to be pathogenic isolates with our trained model increased over time, from 23% in the collection from 1980 to 31% in 2000, and then to 46% in the collection from 2010 (**S5 Fig**). Even though sample storage issues may slightly alter the predictions for samples from 1980 (**S6 Fig, panel B**), we see an even higher increase between 2000 and 2010. The signal of increased pathogenicity would be worth replicating in independent datasets. In fact, applying this predictive model to the large collection of available *E*. *coli* genome sequences, which currently numbers to more than 200,000 genomes [86], could unravel the dynamics of pathogenicity across time and space. One would need to focus on collections with homogenous collection strategies, time and geographic information, and ideally more detailed metadata such as portal of entry—unfortunately such collections remain very rare. This effort would further need to be properly controlled for the biases in the isolates sampled and sequenced (most of them coming from infections), and the phylogroup-specific performance of the model.

What selective pressures might act on pathogenicity determinants? The capacity to cause extra-intestinal infection may not be selected *per se*, as infections are a relatively rare occurrence in the life cycle of *E*. *coli* and do not obviously confer a transmission advantage. Pathogenicity determinants have diverse functions and may therefore be selected for a variety of reasons. They may for example improve the ability to colonize the human gut, improve the ability to compete and replace existing strains, or allow longer persistence in the gut [87–90]. In addition, epistatic interactions between these determinants (**Fig 3G–3J**) and the genetic background of the strains may determine pathogenicity, as recently reported for iron capture systems for virulence in a mouse model [91]. Elucidating the selective pressures acting on these determinants is an important research question that would improve our understanding of *E*. *coli* pathogenicity.

This work opens perspectives to improve studies of the determinants of *E*. *coli* pathogenicity. First of all, genes identified as good pathogenicity candidates not previously reported in *E*. *coli* (*mltC*, *ompX*, etc.) should be validated experimentally in animal models by gene inactivation assays. Second, it remains difficult to pinpoint individual variants because of the clonal structure of *E*. *coli*, and confounding by host factors is a concern. One idea to alleviate clonal structure is to focus on specific STs. This would limit the dominant effect of STs belonging to phylogroup B2 and carrying many virulence genes. However, the genetic diversity within a single ST might also be limited. This makes it difficult to anticipate the results of such ST-focused studies. Another idea is to extend to whole genomes the line of work comparing strains from infections vs. colonization in the same individuals. The case-crossover design reduces concerns on confounding host factors. However, its power is contingent on the within-host diversity of strains present in colonization (**S1 Fig**). In addition, it is difficult in practice to perform a rectal swab in patients arriving at the emergency room for a suspicion of *E*. *coli* BSI and before any antibiotic is prescribed. Third, further help will also likely come from linking pathogen diversity to clinical and epidemiological phenotypes and including the genetic variation of the host into the association such as in a previous study of *S*. *pneumoniae* [70]. Lastly, similar studies should be conducted in low and middle-income countries, where a potentially very distinct diversity of *E*. *coli* circulates [11] and where the public health problem posed by BSI will escalate with the aging population in the years to come.

In conclusion, we elucidated in a systematic and quantitative manner the bacterial genetic determinants of pathogenicity of the major human pathogen *E*. *coli*. The capacity to cause BSI, particularly with urinary PE, is strongly determined by sequence types, additional genetic factors, and tens of specific variants. This implies that *E*. *coli* pathogenicity may evolve, informs future studies of *E*. *coli* mechanisms of pathogenicity, and opens the possibility to reduce the burden of *E*. *coli* with a vaccine targeted at these variants.

## Material and methods

### Ethics statement

All multicenter clinical trials were approved by the appropriate ethic committees. The Colibafi study was approved by the French Comité de Protection des Personnes of Hôpital Saint-Louis, Paris, France (approval #2004–06, June 2004). The Septicoli study was approved by the French Comité de Protection des Personnes Ile de France n°IV (IRB 00003835, March 2016). Because of their non-interventional nature, only an oral consent from patients was requested under French Law. The study on the commensal strains was approved by the ethics evaluation committee of Institut National de la Santé et de la Recherche Médicale (INSERM) (CCTIRS no. 09.243, CNIL no. 909277, and CQI no. 01–014).

### Strain collections

We studied the whole genomes of 1282 *E*. *coli* strains divided in two datasets, 370 commensals strains and 912 BSI strains. Commensal strains were gathered from stools of 370 healthy adults living in the Paris area or Brittany (both locations in the Northern part of France) between 2000 to 2017. These strains come from five previously published collections: ROAR in 2000 (n = 50) [92] (Brittany–a region in the North-West of France), LBC in 2001 (n = 27) [93] (Brittany), PAR in 2002 (n = 27) [93] (Paris area), Coliville in 2010 (n = 246) [94] (Paris area) and CEREMI in 2017 (n = 20) [95] (Paris area) (**S7 Table**). In addition, a collection of 53 commensal strains sampled in 1980 from 53 healthy subjects in Paris (VDG collection) [49] was used to assess the temporal trend of pathogenicity. BSI isolates (Colibafi (n = 367) and Septicoli (n = 545) collections) were collected in 2005 and 2016–2017, respectively [96]. In all studies, one single *E*. *coli* colony randomly picked was retained per individual after plating the blood cultures on non-selective rich medium, or the stools on Drigalsky plates. After this first step, the protocol for all isolates was similar except for the collection from 1980. After one or two subcultures in rich medium, the strains were immediately stored with glycerol at -80°C. The 1980’s collection was stored in agar tubes left at room temperature until the beginning of the 2000s, when the strains were subcultured and stored with glycerol at -80°C.

For the collection of the commensal strains, all participants lived in the community and volunteered to self-collect a faecal swab sample. The inclusion criteria were: age of 18 years or more, no history of gastrointestinal disease, no symptoms of immunosuppression, no antibiotic therapy in the previous month and no hospitalisation in the 3 months preceding inclusion.

The Colibafi study was performed in eight hospitals representing a total of 3,900 adult acute care beds, whereas seven hospitals were included in the Septicoli study, accounting for 5,800 acute care beds. Four hospitals were common between the two studies (i.e. 2,900 acute care beds). All the hospitals belong to the same institution, the “Assistance Publique-Hôpitaux de Paris” network, which accounts for a total of 13,000 adult acute care beds with a homogenous management for most bacterial infections. Clinical data were prospectively collected by clinicians in each center on two separate visits: Visit 1 corresponded to the time of BSI (the day the blood culture was drawn; data were collected retrospectively 24-48h hours later, once the blood culture had grown) and Visit 2 corresponded to the day of discharge or in-hospital death (or day 28 if the patient was still hospitalized). For each episode, the first *E*. *coli* strain collected in the blood culture was identified. The primary endpoint was vital status at discharge or day 28 (i.e. Visit 2). The likely portal of entry was established according to clinical and/or radiological characteristics of the episodes and the isolation of *E*. *coli* from the presumed source of infection. When *E*. *coli* could be isolated from the source of infection, the portal of entry was assigned on the basis of firm clinical suspicion [97]. In each centre, an infectious diseases clinician and a microbiologist were in charge of including patients and completing the case report form (see Colibafi and Septicoli groups in S1 Text). A steering committee was in charge of implementation and a scientific committee responsible for scientific overview.

All the sequences were available (Bioproject PRJEB38489 (ROAR), PRJEB44819 (LBC), PRJEB44872 (PAR), PRJEB39252 (Coliville), PRJEB39260 (Colibafi), PRJEB35745 (Septicoli) and PRJEB44873 (VDG)) except the 20 samples of the CEREMI collection that were whole-genome sequenced in the present work, following the protocol detailed in [31] (Bioproject PRJEB55584).

### Computing the power of case-control and case-crossover studies

We used simulations to compute the power of our case-control study, and compare it to the power achieved with a case-crossover study. We modeled a bacterial genetic variant increasing pathogenicity by +30% (**S1 Fig**). Several strains may independently colonize the gut of individuals. The BSI is caused by a single bacterial strain invading the blood, selected at random from the gut with weight proportional to strain pathogenicity. For simplicity, we assumed all individuals carry the same number of strains.

We first measured the effect of this variant in a simulated study of 912 cases and 370 distinct controls using logistic regression. In the case-control design, the bacterial genetic variant is measured in the strain from BSI and in one randomly chosen strain from the stool samples of controls. Next, we examined a case-crossover design of 912 cases and 912 controls, with controls consisting of one strain randomly chosen from the stool samples of the 912 cases.

We computed both the power to detect this variant across 1000 replicates, and the estimated effect size of the variant. We varied two factors: (i) the number of bacterial strains carried by each host, from 1 to 100. (ii) The presence of a confounding factor that we cannot measure, affecting both the genetic variant and the incidence of infection. The confounding factor is binary, and the variant is present in 30% of hosts with low incidence and 70% of hosts with high incidence. We assumed several strengths of confounding: no confounding, +10% increase in infection, +100% increase in infection.

### Genomic diversity of the core genome

The 1282 assemblies were annotated with Prokka v1.14.6 [98]. We then performed pan-genome analysis from annotated assemblies with Panaroo v1.3.0 with strict clean mode and the removal of invalid genes [99]. We generated a core genome alignment spanning the whole set of core genes as determined by Panaroo, and a phylogenetic tree was computed using FastTree v2.1.11 [100] and visualized using Microreact [101].

### Comparison of commensal and BSI *E*. *coli* collection

Multilocus sequence typing (MLST) was performed using an in-house script Petanc, that integrates several existing bacterial genomic tools [102]. We determined STs (Warwick MLST scheme) [103] and O types [104].

We evaluated the risk of infection associated to colonization by a specific ST and by a specific O-group. We compared the ST and O-group diversity from the collection of 912 BSI isolates with the 370 commensal isolates, for all STs with at least 5 strains in at least one of the two collections and for all O-groups with at least 5 strains in at least one of the two collections.

The odds ratios for the infection risk were computed by fitting a logistic model of infection status (commensal or BSI) as a function of the ST or the O-group (here and thereafter, “significant” refers to significance at the 0.05 level).

Next, we compared the phylogenetic distribution of the commensal collection with the BSI collection. For all strains, we calculated the cumulative frequency distribution of STs in the commensal collection, and we compared it to the same distribution in 200 random sub-samples of 370 sequences from the BSI collection.

We plotted the pangenome variation with the number of genomes analyzed (Panaroo output). We evaluated the pangenome variation between commensal and BSI isolates with Panstripe [105] using the output of FastTree (phylogeny of all strains) and Panaroo (gene presence absence matrix). We randomly subsampled 100 trees of 370 tips from the BSI phylogeny (n = 912) and compared the rate of gene gain and loss between those trees and the commensal tree (n = 370). To quantify the genetic diversity, we computed the pairwise nucleotide diversity (π) [106] in R (package ape) [107].

We also compared the number of virulence factors and the proportion of resistance strains between commensal and BSI isolates. We evaluated the number of VAGs for each of the six main functional classes (adhesin, invasin, iron acquisition, miscellaneous, protectin and toxin) and predicted phenotypic resistance as described in [96] for eight antibiotics of clinical importance (amikacin, ampicillin, cefotaxime/ceftazidime, cefepime, fluoroquinolones, gentamicin, cotrimoxazole and piperacillin/tazobactam). We excluded the resistance to carbapenems in this study because it was very rare (2 strains over 1282). The odds ratios for the infection risk were computed by fitting a logistic model of infection status (commensal or BSI) as a function of the number of VAGs or the status of resistance (resistant versus sensitive).

We evaluated the co-occurrences of the four major iron-capture systems and of the four major adhesins systems (complete genes or alleles), defined as the systems with a significance < 10^−7^ when comparing commensals against BSI strains (see **S4 Table**). The iron capture systems (HPI, operon *iro*, *iuc* and *sit*) and the adhesin systems (*ecp* and *pap*) were considered present when all their genes were detected, at the exception of *papA* which is very rare and *papG* for which we examined the allele *papGII* (see below). We detected the presence of genes with Abricate [108] with 75% identity and 50% coverage. The adhesin alleles, *papGII* and *iha7*, were detected with Abricate with 90% identity and 90% coverage. We performed a lasso regression to select the genes and/or alleles that best predict the pathogenicity among the four adhesins systems, the four iron capture systems and the eight systems simultaneously. We estimated the CIs with 1000 bootstrap replicates.

### Heritability estimates

We estimated narrow-sense heritability for the target variable using 2 different covariance matrices: one built from the genetic variants using a kinship matrix, and another one with the sequence types membership. Limix v3.04 [109] was used, assuming normal errors for the point estimate.

### Association analysis

We derived unitigs using unitig-counter v1.1.0 [43]. We tested locus effects using the wg (whole genome) model of pyseer v1.3.6 [42,110], which trains a linear model with elastic net regularization using the presence/absence patterns of all unitigs. We used the parameter alpha with value of 1 for the elastic net, which is equivalent to a lasso model. The model performance was assessed by computing three metrics using each phylogroup. The precision, as the measure of how many positive predictions made are correct; the recall, as the measure of how many positive cases the classifier predicted correctly over all the positive cases; and the F1-score, as the harmonic mean of the two metrics. The F1-score expected by chance was computed overall, for each phylogroup and for the different subsets, by randomly assigning the phenotype to the test samples and running 1000 randomizations. The unitigs with a non-zero model coefficient were mapped back to all input genomes, and gene families were annotated by taking a representative protein sequence from all genomes encoding each gene, which was then used as the input for eggnog-mapper v2.1.3 using the panaroo output to collapse gene hits to individual groups of orthologs. Genes downstream and upstream of unitig hits within intergenic regions were further mapped back. GO terms enrichment was determined using goatools v1.2.3 [111]. An *in-house* list of *E*. *coli* virulence genes and antibiotic resistance genes was used to annotate the virulence and antibiotic resistant genes within the collection, and a Fisher’s exact test was used to determine the enriched genes, with a multiple testing correction based on the Benjamini-Hochberg method, with a 5% family-wise error rate. For the COG and virulence genes enrichment analysis a random ST131 genome from the full dataset was picked up as background.

### Prediction analysis

We used unitig-caller v1.3.0 [112] to make variant calls in the test population, and the elastic net regularization, previously trained, model using pyseer v1.3.6 [110] to predict the phenotype in new commensal samples from different time periods, divided in decades.

### Genome sequence quality control and storage effect assessment

To quantify general genome assembly quality, we used the N50 metric, defined as the length of the shortest contig at half the total length of the assembly. A smaller N50 value indicates a genome assembly with shorter contigs.

We used the presence of genetic variants in the *rpoS* gene as a metric for appropriate sample storage between isolation and sequencing; it has been shown that repeated freeze/thaw cycles and long-term storage in agar stabs induce mutations in this gene, as well as a possible marker for deletions [50]. We used snippy v4.6.0 to call and annotate variants between each genome assembly and the *E*. *coli* str. K-12 *substr.* MG1655 reference (RefSeq NC_000913.3). We filtered out synonymous variants as well as one common non-synonymous variant (Gln33Glu) and counted the remaining ones for each sample.

For the oldest collection (VDG, 1980), we also compared the results of the typing for the presence of the *hly* and *pap* genes assessed phenotypically (production of alpha hemolysin using horse erythrocyte agar plates and presence of mannose-resistant hemagglutination using glass microscope slides [113]) and genetically (PCR method for *hlyC* and *papC* detection) performed in 1980 [51] and 2000 [49], respectively.

Lastly, we checked whether gene loss in older collections could have resulted in the loss of genes involved in virulence, and therefore bias pathogenicity downwards in the sequences from 1980. To first examine potential trends in gene content, we selected the genes present in 5 to 95% of the strains from 1980 to 2010 (total 4895 genes). For 100 randomly chosen genes, we used a linear model to assess the association between gene frequency and sampling date. The mean effect size was +0.0012 per year (+3.5% frequency over 30 years). This trend could be due to the loss of genes in older collections (possible if the bacteria replicate slowly, in particular in stab culture and for plasmid-borne genes), but also to changes in the phylogenetic composition of the population. To assess how this slight trend could have changed pathogenicity, we first identified the unitigs selected in the GWAS that reflect gene presence/absence. The criterion was a correlation > 0.5 between gene presence/absence and unitig presence/absence. 13 unitigs out of 59 met this criterion. We then simulated two synthetic datasets of size 10000, one reflecting our main dataset (2000–2010) and one reflecting a hypothetical dataset of 1980 affected by the -3.5% frequency of the 13 genes identified selected in the GWAS. This was done by randomly drawing unitig presence/absence for each sequence with the corresponding frequency, and neglecting linkage. We predicted that the synthetic dataset of 1980 counted 27.5% of commensals, and that of 2000–2010 27.1% of commensals. Thus, a bias in gene content in 1980 could not possibly have caused the observed trends in pathogenicity, both because the observed trend in gene content is weak, and because the GWAS model does not predict a strong net positive effect of more genes on pathogenicity.

### Code availability

Apart from the software packages mentioned in the previous sections, the following were used to run the analysis and generate the visualizations presented in this work: pandas v1.3.4 [114], numpy v1.20.3 [114], scipy v1.7.1 [115], matplotlib v3.4.3 [116], seaborn v0.11.2 [117], biopython v1.80 [118] jupyterlab v3.2.1 [119]. Most of the analysis were incorporated in a reproducible pipeline using snakemake v7.18.1 [120] and conda v4.10.3 [121], which is available as a code repository on GitHub (https://github.com/jburgaya/2022_ecoli_commensal) under a permissive licence (MIT).

## Supporting information

S1 Table Distribution of the phylogroups of the *E*. *coli* commensal and BSI collections isolates for all phylogroups present in at least 5 strains in at least one of the two collections. (XLSX)

S2 Table Distribution of the sequence types of the *E*. *coli* commensal and BSI collections isolates. The number of isolates and the percentage are presented in the table. We compared the ST diversity of *E*. *coli* isolates from BSI (all portals of entry, urinary portal of entry and digestive portal of entry) with a collection of commensal isolates, for all STs present in at least 5 strains in at least one of the two collections. We show the odds ratio (with 95% CI) for the risk of infection associated with colonization by each ST (logistic model of infection status as a function of the ST). STs with odds ratio significantly different from 1 are highlighted in bold.
(XLSX)

S3 Table Distribution of the O-groups of the *E*. *coli* commensal and BSI collections isolates. The number of isolates and the percentage are presented in the table. We compared the O-group diversity of *E*. *coli* isolates from BSI (all portals of entry, urinary portal of entry and digestive portal of entry) with the collection of commensal isolates, for all O-groups present in at least 5 strains in at least one of the two collections. We show the odds ratio (with 95% CI) for the risk of infection associated with colonization by each O-group (logistic model of infection status as a function of the O-group). O-groups with odds ratio significantly different from 1 are highlighted in bold.
(XLSX)

S4 Table Effect sizes (and 95%CI) of the comparison of the number of VAGs between commensal and BSI strains for the six main functional classes of virulence As suggested by Cohen (1988) effect sizes are negligible under 0.2 (in gray), small between 0.2 and 0.5 (in blue), medium between 0.5 and 0.8 (in yellow) and large above 0.8 (in red). (XLSX)

S5 Table Comparison of the distribution of virulence associated genes (VAGs) between commensal and BSI strains. VAG proportions of commensal and BSI strains are indicated between brackets. Significant differences are in bold (at the 0.05 level).
(XLSX)

S6 Table Genes to which unitigs with non-zero model weights mapped to them. Genes are ordered by their average LRT pvalue, annotation columns are derived from the eggnog-mapper.
(XLSX)

S7 Table GO term enrichment for the genes with unitigs mapped to them (S6 Table). (XLSX)

S8 Table Recapitulative table of the typing analyses (petanc) and of the strain sampling characteristics. Phylogroups, MLSTs (Warwick and Pasteur), serotypes and fimH alleles are indicated.
(XLSX)

S9 Table Genes upstream and downstream of the unitigs only found in intergenic regions, to which unitigs with non-zero model weights mapped to them. Genes are ordered by their average LRT pvalue, annotation columns are derived from the eggnog-mapper.
(XLSX)

S1 Fig Power of the case-control (blue; 912 BSI against 370 commensals) and case-crossover (red; 912 BSI against 912 commensals from the same individuals) designs to detect the effect of a bacterial genetic variant increasing pathogenicity by +30% (top row), and inferred effect (bottom row). This is shown for three strengths of confounding: no confounding (left), weak confounding (middle; +10% increase in incidence of infection), strong confounding (right; +100% increase in incidence of infection). The case-control study consistently has better power but is subject to over-estimating the effect size of a variant because of confounding. The case-crossover study has very poor power when the number of strains is small, maintains the same power regardless of confounding, and can estimate properly the effect size with large number of colonizing strains. The case cross-over study can have higher power than the case-control study in the absence of confounding (top left graph) bcause it benefits from a larger sample size (912+912).
(PNG)

S2 Fig (A-C) Comparison of the distribution of VAGs per strain among the six main functional classes of virulence of the *E*. *coli* commensal and BSI collections isolates. We show the odds ratio (OR with 95% CI) for the risk of infection associated with the number of VAGs (logistic model of infection status as a function of the number of VAGs), for (A) all the B2 strains (467 BSI strains and 120 commensal strains), (B) B2 BSI strains with urinary portal of entry to B2 commensals (304 BSI strains) and (C) B2 BSI strains with digestive portal of entry to B2 commensals (124 BSI strains). Functional classes of virulence are ordered by increasing associated odds ratio for all B2 strains.
(PNG)

S3 Fig (A-D) Comparison of the distribution of resistant strains for eight antibiotics of clinical importance of the *E*. *coli* commensal and BSI collections isolates. We show the odds ratio (OR with 95% CI) for the risk of infection associated with the resistance of strains (logistic model of infection status as a function of the resistance of strains), for (A) all the B2 strains (467 BSI strains and 120 commensal strains), (B) B2 BSI strains with urinary portal of entry to B2 commensals (304 BSI strains), (C) B2 BSI strains with digestive portal of entry to B2 commensals (124 BSI strains) and (D) BSI Colibafi strains (sampled in 2005) to commensals (367 BSI strains). Categories of antibiotics are ordered by increasing associated odds ratio for all B2 strains. For AMK, FQ, GEN and FEP categories, we only show the lower bound of the CI because the estimated odds ratios are huge as none of the commensal isolates were resistant to these antibiotics. AMK, amikacin; AMP, ampicillin; CTX/ CAZ, cefotaxime/ceftazidime; FEP, cefepime; FQ, fluoroquinolones; GEN, gentamicin; SXT, cotrimoxazole; TZP, piperacillin/tazobactam.
(PNG)

S4 Fig wg-GWAS model performance within each phylogroup. F1-score representation (blue dots), precision (yellow dots), and recall (red dots). A) For the full collection B) the subset of clinical isolates with urinary tract as portal of entry, and C) the subset of clinical isolates with digestive tract as portal of entry. The naive and the analysis with covariates are represented. PE: portal of entry.
(PNG)

S5 Fig Proportion of BSI predicted isolates over time. 423 isolates from commensal collections were fitted to the trained ML model. The proportion of BSI isolates for the 3 different periods of time is colored in red and the percentage indicated above each bar. The total number of isolates per year is given in brackets.
(PNG)

S6 Fig Quality control of the genome sequences used in this study. A) Genome assembly quality does not differ substantially across strain collections, as measured using the N50 metric. B) Putatively deleterious mutations in rpoS, a metric for sample storage quality is negligible for all collections used for the main analysis, and much higher for the excluded collection, for which we had a lower confidence on sample storage.
(PNG)

S1 Text The names of the collaborators of the Colibafi/Septicoli and Coliville groups. (DOCX)
