# Supplementary figures and images for: The bacterial genetic determinants of Escherichia coli capacity to cause bloodstream infections in humans
Source: PLoS Genet. 2023 Aug 2;19(8):e1010842. doi: 10.1371/journal.pgen.1010842 (PMC10395866; doi:10.1371/journal.pgen.1010842)

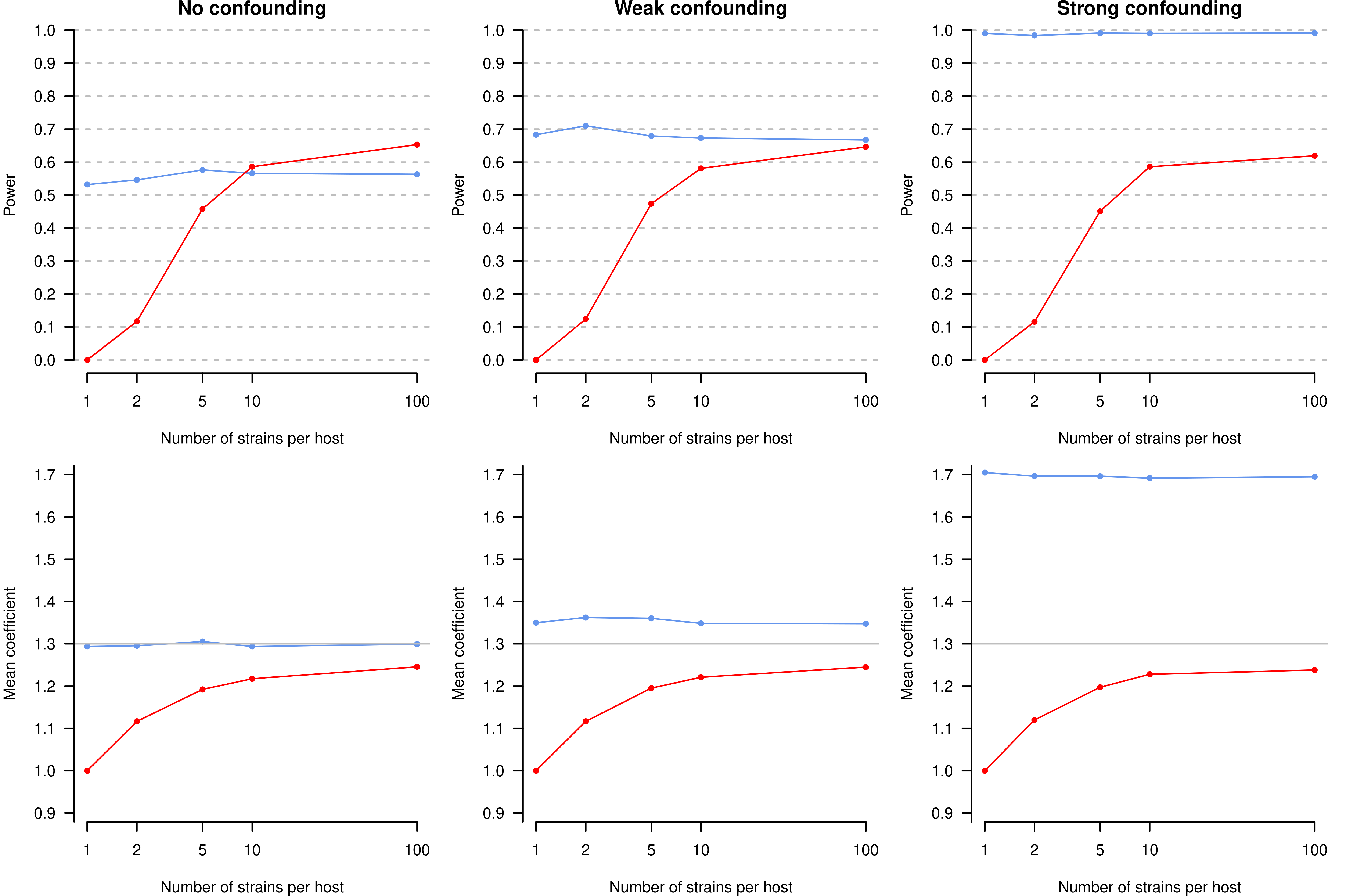

Supplement: S1 Fig — This is shown for three strengths of confounding: no confounding (left), weak confounding (middle; +10% increase in incidence of infection), strong confounding (right; +100% increase in incidence of infection). The case-control study consistently has better power but is subject to over-estimating the effect size of a variant because of confounding. The case-crossover study has very poor power when the number of strains is small, maintains the same power regardless of confounding, and can estimate properly the effect size with large number of colonizing strains. The case cross-over study can have higher power than the case-control study in the absence of confounding (top left graph) bcause it benefits from a larger sample size (912+912). (PNG) [file pgen.1010842.s010.png]

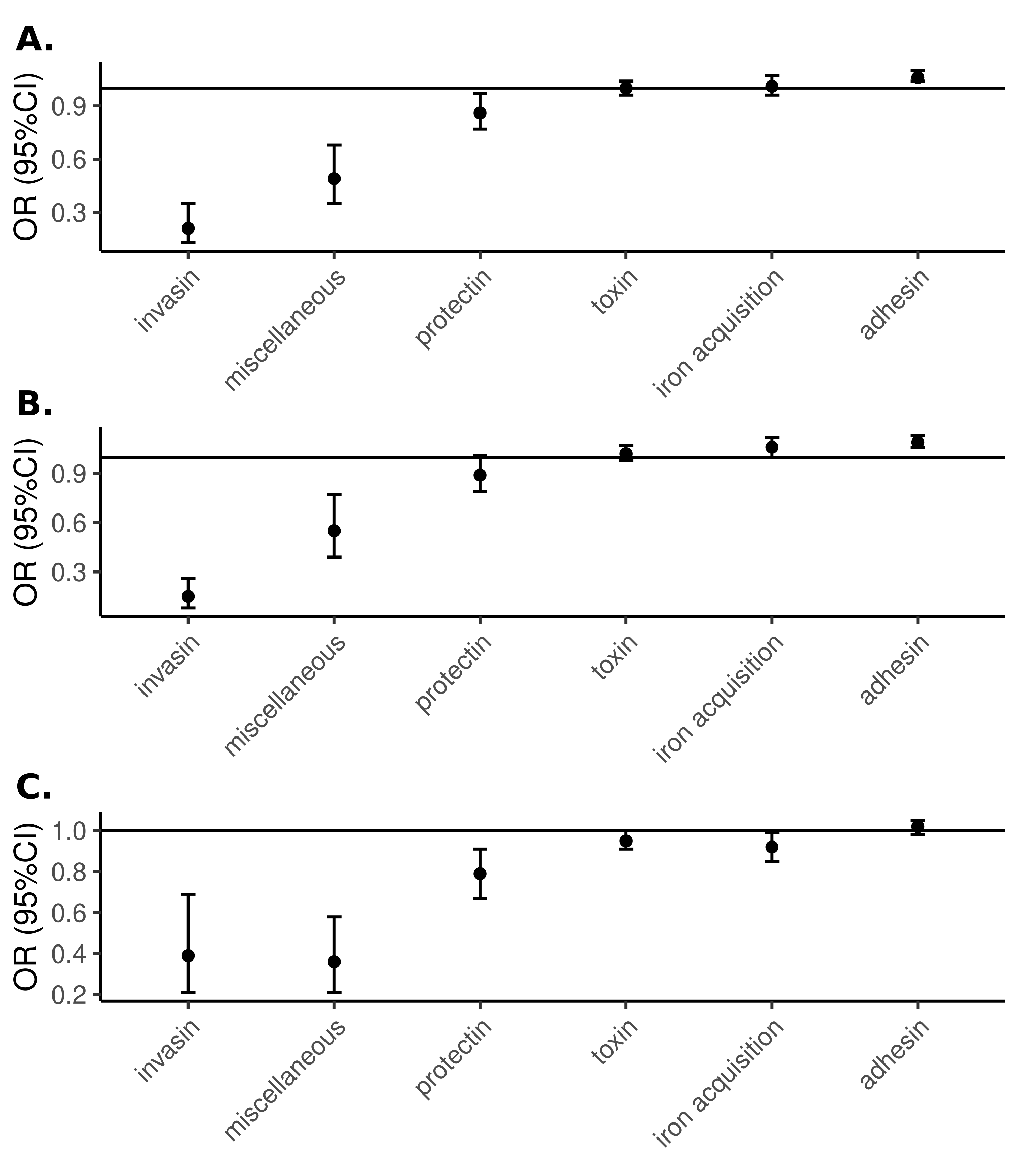

Supplement: S2 Fig — (A-C) Comparison of the distribution of VAGs per strain among the six main functional classes of virulence of the E. coli commensal and BSI collections isolates. We show the odds ratio (OR with 95% CI) for the risk of infection associated with the number of VAGs (logistic model of infection status as a function of the number of VAGs), for (A) all the B2 strains (467 BSI strains and 120 commensal strains), (B) B2 BSI strains with urinary portal of entry to B2 commensals (304 BSI strains) and (C) B2 BSI strains with digestive portal of entry to B2 commensals (124 BSI strains). Functional classes of virulence are ordered by increasing associated odds ratio for all B2 strains. (PNG) [file pgen.1010842.s011.png]

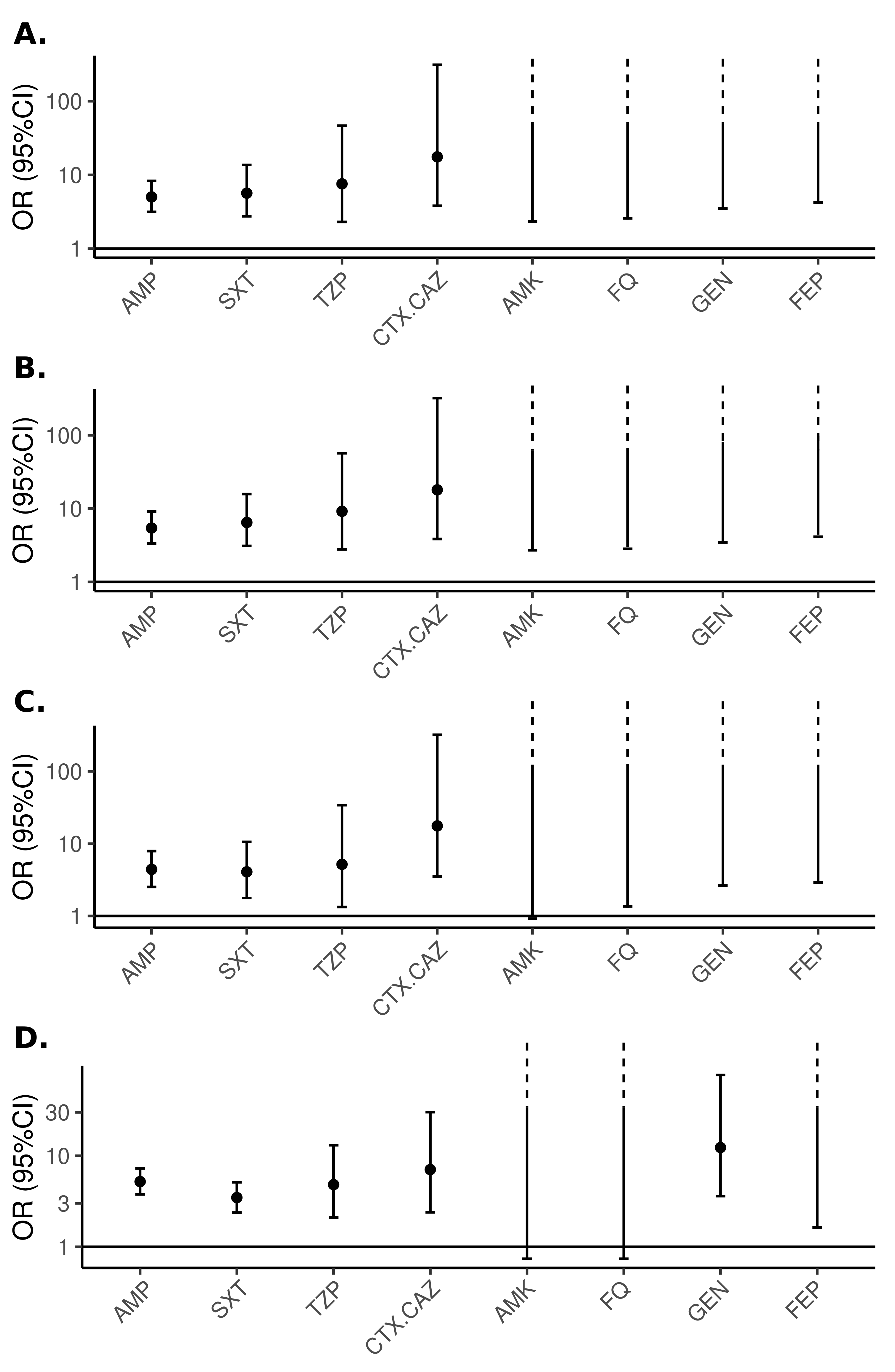

Supplement: S3 Fig — (A-D) Comparison of the distribution of resistant strains for eight antibiotics of clinical importance of the E. coli commensal and BSI collections isolates. We show the odds ratio (OR with 95% CI) for the risk of infection associated with the resistance of strains (logistic model of infection status as a function of the resistance of strains), for (A) all the B2 strains (467 BSI strains and 120 commensal strains), (B) B2 BSI strains with urinary portal of entry to B2 commensals (304 BSI strains), (C) B2 BSI strains with digestive portal of entry to B2 commensals (124 BSI strains) and (D) BSI Colibafi strains (sampled in 2005) to commensals (367 BSI strains). Categories of antibiotics are ordered by increasing associated odds ratio for all B2 strains. For AMK, FQ, GEN and FEP categories, we only show the lower bound of the CI because the estimated odds ratios are huge as none of the commensal isolates were resistant to these antibiotics. AMK, amikacin; AMP, ampicillin; CTX/ CAZ, cefotaxime/ceftazidime; FEP, cefepime; FQ, fluoroquinolones; GEN, gentamicin; SXT, cotrimoxazole; TZP, piperacillin/tazobactam. (PNG) [file pgen.1010842.s012.png]

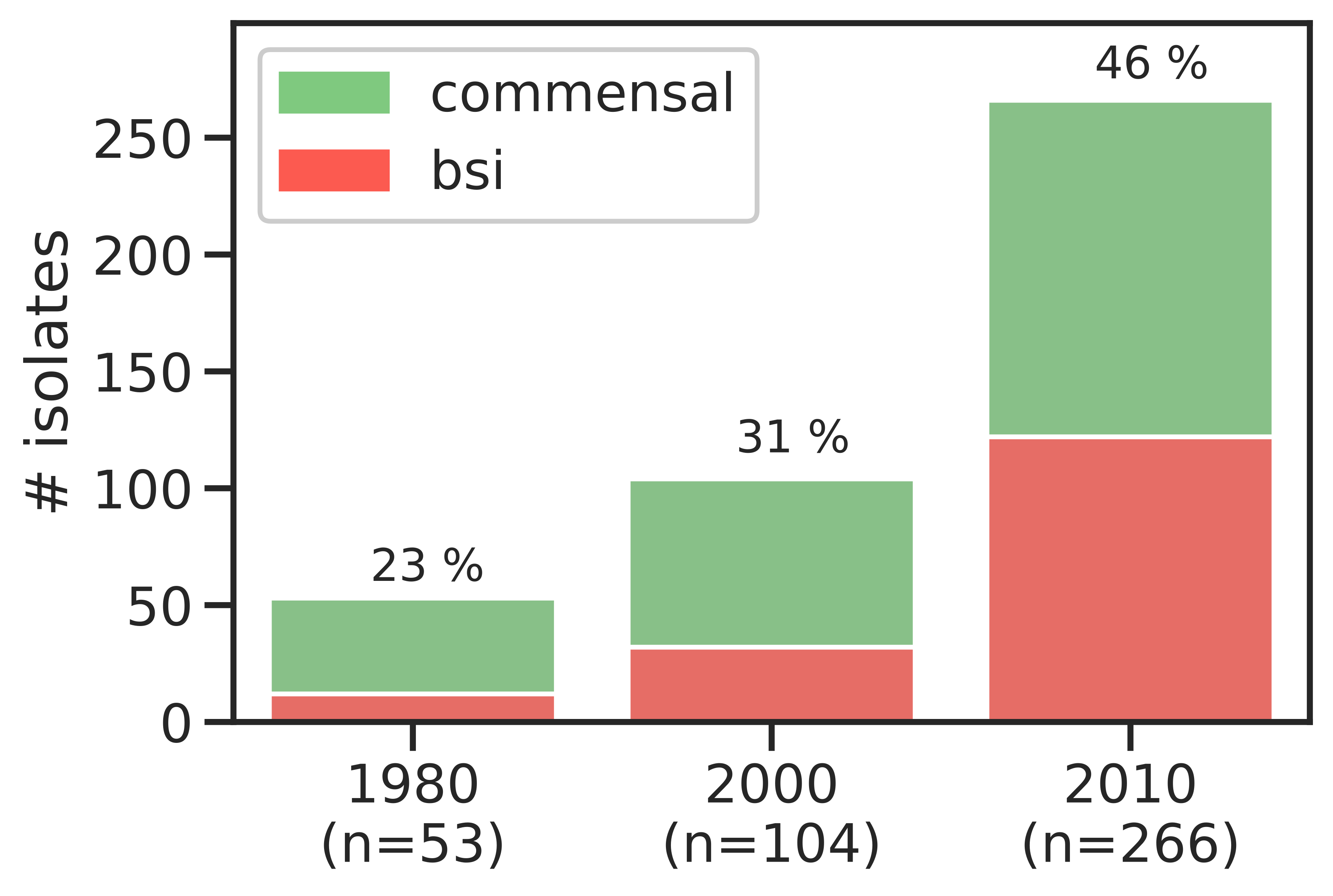

Supplement: S5 Fig — 423 isolates from commensal collections were fitted to the trained ML model. The proportion of BSI isolates for the 3 different periods of time is colored in red and the percentage indicated above each bar. The total number of isolates per year is given in brackets. (PNG) [file pgen.1010842.s014.png]

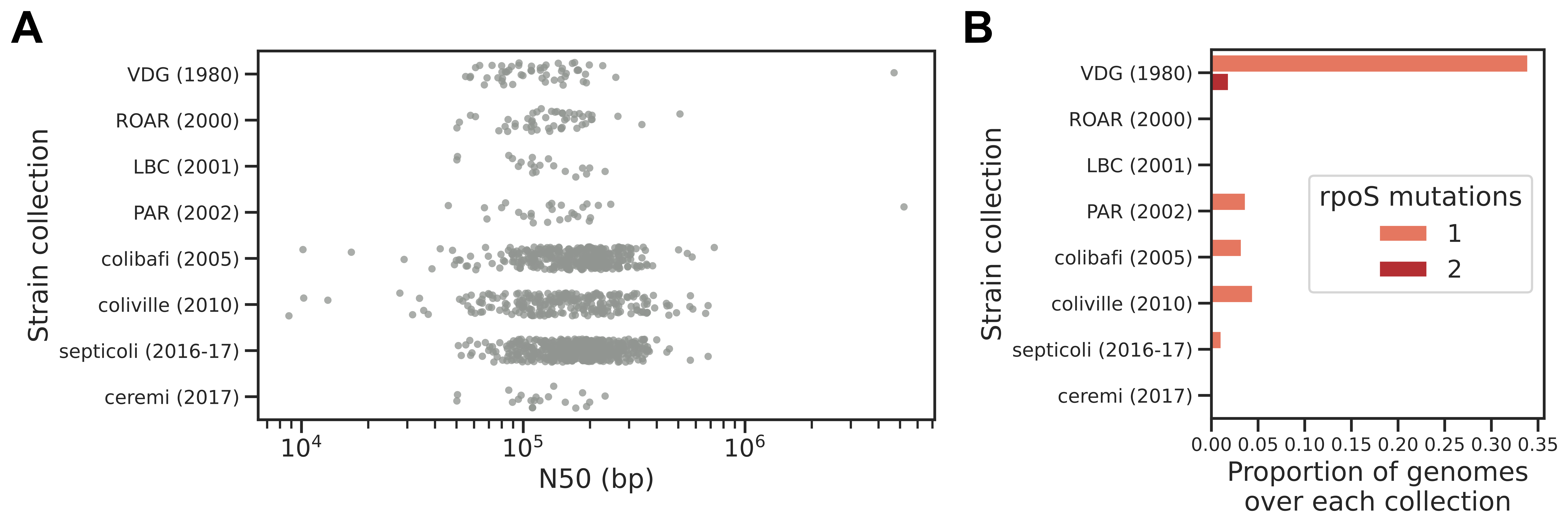

Supplement: S6 Fig — A) Genome assembly quality does not differ substantially across strain collections, as measured using the N50 metric. B) Putatively deleterious mutations in rpoS, a metric for sample storage quality is negligible for all collections used for the main analysis, and much higher for the excluded collection, for which we had a lower confidence on sample storage. (PNG) [file pgen.1010842.s015.png]
