# Supplementary material for: The bacterial genetic determinants of Escherichia coli capacity to cause bloodstream infections in humans
Source: PLoS Genet. 2023 Aug 2;19(8):e1010842. doi: 10.1371/journal.pgen.1010842 (PMC10395866; doi:10.1371/journal.pgen.1010842)
Supplement: S1 Text — (DOCX) [file pgen.1010842.s016.docx]

**The Colibafi/Septicoli group is composed of:** Michel Wolff, Loubna Alavoine, Xavier Duval, David Skurnik, Paul-Louis Woerther, Antoine Andremont, Etienne Carbonnelle, Olivier Lortholary, Xavier Nassif, Sophie Abgrall, Françoise Jaureguy, Bertrand Picard, Véronique Houdouin, Yannick Aujard, Stéphane Bonacorsi, Agnès Meybeck, Guilène Barnaud, Catherine Branger, Agnès Lefort, Bruno Fantin, Claire Bellier, Frédéric Bert, Marie-Hélène Nicolas-Chanoine, Bernard Page, Julie Cremniter, Jean-Louis Gaillard, Françoise Leturdu, Jean-Pierre Sollet, Gaëtan Plantefève, Xavière Panhard, France Mentré, Estelle Marcault, Florence Tubach, Virginie Zarrouk, Frederic Bert, Marion Duprilot, Véronique Leflon-Guibout, Naouale Maataoui, Laurence Armand, Liem Luong Nguyen, Rocco Collarino, Anne-Lise Munier, Hervé Jacquier, Emmanuel Lecorché, Laetitia Coutte, Camille Gomart, Ousser Ahmed Fateh, Luce Landraud, Jonathan Messika, Elisabeth Aslangul, Magdalena Gerin, Alexandre Bleibtreu, Mathilde Lescat, Violaine Walewski, Frederic Mechaï, Marion Dollat, Anne-Claire Maherault, Mélanie Mercier-Darty, Bernadette Basse, Bruno Fantin, Xavier Duval, Etienne Carbonnelle, Jean-Winoc Decousser, Raphaël Lepeule.

**The COLIVILLE group is composed of:** Monique Allouche, Jean-Pierre Aubert, Isabelle Aubin, Ghislaine Audran, Dan Baruch, Philippe Birembaux, Max Budowski, Emilie Chemla, Alain Eddi, Marc Frarier, Eric Galam, Julien Gelly, Serge Joly, Jean-François Millet, Michel Nougairede, Nadja Pillon, Guy Septavaux, Catherine Szwebel, Philippe Vellard, Raymond Wakim, Xavier Watelet and Philippe Zerr.
