# Supplementary material for: The bacterial genetic determinants of Escherichia coli capacity to cause bloodstream infections in humans
Source: PLoS Genet. 2023 Aug 2;19(8):e1010842. doi: 10.1371/journal.pgen.1010842 (PMC10395866; doi:10.1371/journal.pgen.1010842)
Supplement: Attachment — Submitted filename: response_to_reviewers.pdf [file pgen.1010842.s017.pdf]

Dear Editor,

We would like to submit this manuscript for consideration to PLOS Genetics. We submitted this manuscript before to PLOS Biology, and it was rejected. However, we note that the reviewers were overall positive in their assessment of our work: *"This is a really interesting manuscript that describes a detailed phylogenomic analysis of E. coli genomes sourced from humans", "The analyses are conducted in a robust manner and the conclusions are justified", "Such studies could help explain why only some E. coli strains have the ability to cause disease, and could help improve diagnostics and vaccine development efforts", "This paper does add to the field and helps define what it takes for E. coli strains to cause BSI"*. Several of the criticisms of reviewer #2, the most critical of our work, are in our opinion unwarranted, including misunderstanding of the general purpose of our study, and unfounded criticisms on data quality.

We have addressed all reviewers' concerns in a revised version of the manuscript where we run several new analyses. Thanks to the reviewer comments, the manuscript has been clearly improved. We would like to acknowledge the reviewers and to submit this revised manuscript for publication in PLOS Genetics.

This work on pathogenicity in human *E. coli* strains is the third part of our efforts to understand pathogenicity and virulence in *E. coli* using GWAS and natural isolates. Whereas the first part studied virulence in mice (Galardini et al., PLoS genetics, 2020, 16 (10), e1009065, 41 citations in Google Scholar), the second part looked to virulence in humans (Denamur et al., PLoS Genetics, 2022, 18 (3), e1010112, 10 citations in Google Scholar). With this third part, we bring new and complementary data on pathogenicity in humans.

We detail below our responses to reviewers. Major changes compared to the previous version of the manuscript are highlighted in blue in the revised manuscript; all line and figure numbers below refer to the revised manuscript.

PLOS Biology editor's comments

Please allow me to first apologize for the delay in the processing of your manuscript. This delay is caused by my difficulty in recruiting reviewers for your manuscript. I am sorry for this unexpected event, and I thank you for your patience while your manuscript "The bacterial genetic determinants of Escherichia coli capacity to cause bloodstream infections in humans" was peer-reviewed by PLOS Biology. Your work was assessed and discussed by the PLOS Biology editorial team, an Academic Editor with relevant expertise, and by several independent reviewers. Based on the reviews, which you will find at the end of this email, I regret that we will not be pursuing your manuscript for publication in the journal.

As you will see, the reviewers' concerns regarding the lack of experimental validation of the factors found, that the evidence of increase pathogenicity is not well substantiated, and the issues raised by Reviewer #2 regarding the collection used, are of sufficient importance to preclude publication at PLOS Biology.

We responded to these major comments as such (more detailed answers are provided along the reviewers' comments):

Point 1: The lack of experimental validation of the factors found.

This point concerns the newly identified factors such as *mltC*, *ompX* that need experimental validation. The KO and complementation of each gene followed by functional tests in animal models of gut colonization and sepsis represent a considerable amount of work. Moreover, the phenotype we study here (commensalism vs. infection) is not directly measurable experimentally, as infections represent a very rare event. With experimental work, we can access phenotypes such as density in the gut, survival in the urine or blood, or the severity of infection. These phenotypes indirectly and incompletely reflect the different steps from the gut colonization to blood infection. Such indirect evidence is actually already present for these genes, sometimes for other species (as cited Table 1). The experimentation to prove causality of the identified intergenic variants is even more difficult (see below). All in all, we feel such experimental work is outside the scope of the presented work. We add a sentence in the discussion section on this point.

Point 2: The evidence of increased pathogenicity is not well substantiated

We predicted pathogenicity in sequences from 1980, and found pathogenicity at that time was lower than in 2000 and 2010. In our opinion, the main weakness of this result is actually that it concerns the temporal dynamics <u>in France only</u>, and it is unknown whether this could be extrapolated to other countries. However, as *E. coli* causes a major burden especially in Western countries, this signal is sufficiently important to be highlighted as an important result. Reviewer 3 raised two specific concerns, contamination and loss of genes including VAG due to collection issues in the collection from 1980. The samples from 1980 were stored in stab from 1980 to 2000. We emphasize that the collection and conservation schemes were similar for all collections from 2000 onwards. After 2000, all samples were stored at -80 with only one or two cultivation steps between the stool and the stored sample. We checked the robustness of our result to potential biases in collection in four ways:

1) We checked whether there was any systematic trend in genome quality, measured with the widely accepted N50 metric reflecting the length of contigs (Supp. Figure 6A, reproduced below) between 1980 and later time-points and did not find any trend.

2) We further checked for mutations in the *rpoS* gene as a marker of sample degradation (doi: 10.1128/JB.01972-14) and found that less than 5% of samples from all the collections used to train the machine learning GWAS model had putatively deleterious mutations in this gene (Supp. Figure 6B, reproduced below). We however found that for our strain collection from 1980 up to almost 35% of strains had at least one putatively deleterious variant in *rpoS*, which further reinforces our initial decision to exclude this collection from the model training.

3) To assess potential gene loss in collections from 1980, we compared results on the presence/absence of the two genes (*hly* and *pap*) in our 53 whole-genome sequences from 1980 to previous studies on the same collection. The Hly and mannose-resistant haemagglutination (MHRA) phenotypes reflecting the presence of the *hly* and *pap* genes were at frequency 5% and 7% respectively (as measured in 1983, doi: 10.1093/infdis/154.4.727). The small differences observed as compared to the WGS data may be explained by the imperfect phenotype/genotype correlation. Nevertheless, phenotypically assessed, these strains had a very low level of VAGs. The presence of *pap* and *hly* genes was also assayed by PCR in 1999/2000, with a perfect match with results based on the recently-generated whole genomes (doi: 10.1099/00221287-147-6-1671, Table 3). These two genes thus do not appear to have been lost over time.

4) We investigated whether there was any systematic trend in all genes presence/absence from 1980 to 2010 and how this could affect predicted pathogenicity. Although we did find a light trend (which could be explained both by spurious loss of genes in old samples, or changes in phylogenetic composition), this trend did not result in any effect on pathogenicity both because it was small and because there was no strong net positive effect of more genes on pathogenicity (Results lines 353-371, Material and Methods line 685).

Lastly, we note that the predicted increase between 2000 and 2010 would not be affected by sample degradation noted in (2). We have therefore left this analysis in the revised paper as a potentially interesting observation for others to replicate in similar high quality and time-resolved collections. We carefully reworded the corresponding sections to reflect the uncertainty.

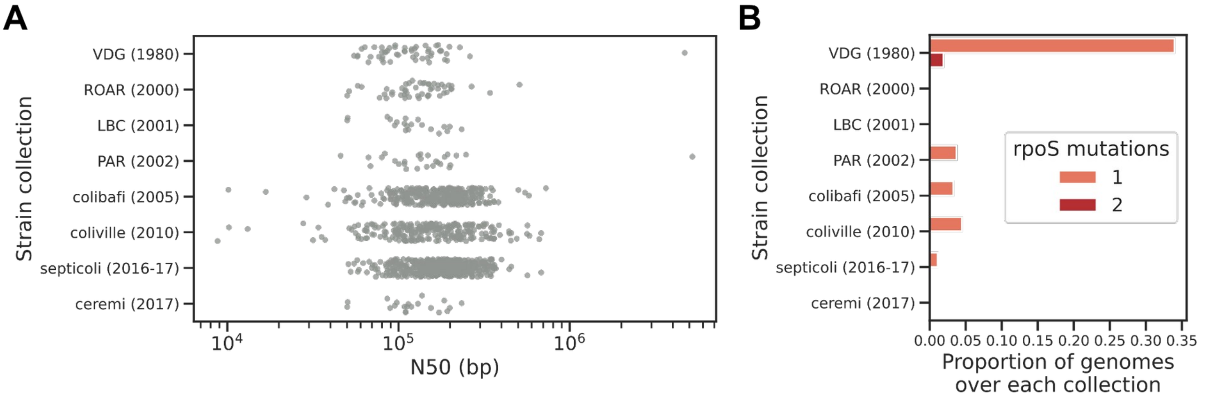

Supplementary figure 6: Quality control of the genomes used in this study. A) Genome assembly quality does not differ substantially across strain collections, as measured using the N50 metric. B) Putatively deleterious mutations in *rpoS*, a metric for sample storage quality is negligible for all collections used for the main analysis, and much higher for the 1980 collection, for which we had a lower confidence on sample storage.

Point 3: The concerns on the collection used.

We think these concerns are not warranted. Both BSI and commensal samples were collected in a 17-year time-period as stated clearly in the material and methods

section. The statement “and they come from different countries” is incorrect and surprising, considering that we clearly wrote “collected in France”. To clarify, Brittany is a region in the North West of France (Bretagne), not a different country.

Lastly, we also detail better the clinical data to explain how we determined the portal of entry (see material and methods). We explained better how all strains were isolated and stored: a similar protocol was used for all blood samples, and all stool samples (contrary to the claim of reviewer 2).

Point 4: The originality of the work

We add here a point of response to a comment from reviewer 3 questioning the originality of our work. This point was not mentioned by the editor as a major concern, but we think it is important to clarify how our work is novel. We emphasize that this is the **first study with genome-wide comparison of commensal and BSI strains in *E. coli***. Our main results are the large heritability of pathogenicity, the findings of determinants of pathogenicity independently of the genetic background, and some indications that pathogenicity might have increased in France over the past decades. All these findings are novel compared to previous work from our groups and others. An increasing number of GWAS investigating the pathogenicity of various bacterial species have been published, with better methods and larger genomic collections, as reviewed in the Discussion. Ours is the first for *E. coli*, one of the most important bacterial pathogens and one of the few species classified as Group B: ”*pathogens with vaccines that are in late-stage clinical trials with high development feasibility”* (WHO technical document on bacterial vaccines, available at https://www.who.int/publications/i/item/9789240052451). This is the group for which studies such as ours are most relevant.

> **Reviewer #1:**
>
> Reviewer #1: This is a really interesting manuscript that describes a detailed phylogenomic analysis of E. coli genomes sourced from humans (healthy commensal E. coli) and patients with blood stream infections (BSI). The analyses are conducted in a robust manner and the conclusions are justified. I only found minor efforts and provide some suggestions for improving the manuscript. Overall it is well written with only minor typographic errors (see minor points).

Thank you for these comments.

> Major points.
> Line 33/34 It would be good to provide the numbers in the abstract (X BSI; Y commensal)

Done in the abstract.

> Line 312. "This is roughly double of the heritability when considering STs alone, suggesting that specific genetic variants at a finer phylogenetic scale than ST are determining pathogenicity." While ST is useful there is a lot of lineage-specific

information in regards to pathogenicity that is not captured when one refers to a ST. This has been demonstrated in ST95 (PMID: 35076267) and in ST372 (PMID: 36752777).

Yes precisely, this is one of our important points. We highlight this point in the discussion (around lines 424 and 514).

Line 345 The authors state "This suggests that antibiotic resistance genes are genetically linked with pathogenicity determinants, and opens the interesting possibility that antibiotic resistance coevolves with pathogenicity determinants associated with the clonal background of E. coli"
This is not a novel concept and has been discussed in numerous papers. There are plenty of plasmids that carry both AMR and important virulence gene cargo. Most recently this is exemplified in the phylogenetic evolution of ST1193 (PMID: 36409140), but also in ColV F virulence plasmids (PMID: 29966679) but also in papers going back to 2010 in enterohaemorrhagic E. coli (PMID: 19917674). Importantly is also reported in the evolution of pandemic lineages of Salmonella enterica serovar Typhimurium where a genomic island known as Salmonella genomic Island 1 (PMID: 16713724; PMID: 22125606) comprises a combination of virulence gene cargo and AMR genes. SGI1 and variants are now known to be dispersed more widely than in Salmonella (PMID: 31979280) including E. coli (PMID: 31118300). I add this information simply to demonstrate the point.

We would like to clarify our findings in relation to the reviewer statement.

The references are indeed relevant: convergence of resistance and virulence on the same genetic element is an indication that AMR and virulence might be co-selected, and we now mention this at lines 473-474 of the Discussion. Our findings concur in suggesting that AMR and virulence are co-selected, but the nature of the evidence is quite distinct.

We find that while AMR genes are over-represented in BSI isolates compared to commensal isolates, only one AMR gene is associated with BSI when controlling for population structure. Our conclusions are (i) AMR does not have a direct causal role in virulence and (ii) AMR may be associated with virulence genes or virulent STs. Conclusion (i) can be reached by comparison of commensal and BSI collections, not by examination of BSI genomes alone. Conclusion (ii) is similar to the conclusion emerging from the discovery of AMR-virulent plasmids, but again the evidence to reach the conclusion is distinct.

The strengths of our analysis are the following: we exclude a causal role of most AMR in infection, we use the whole phylogenomic diversity to reach our conclusions, and we detect co-evolution even between elements that are not necessarily linked on the same chromosomal region or plasmid. The properties of bacterial recombination enable associations to emerge even between physically distant genes (doi: 10.1534/genetics.117.300662). Indeed, we investigated the proximity between VAGs and AMR in our collections, and found that VAGs and AMR genes are never

encoded in the same contig in the genomes used in this study (see also our reply to comment #4 from Reviewer 3).

We now develop this discussion on the convergence of resistance and virulence on the same genetic element, and the link with our findings, at lines 473-479 of the Discussion.

Minor points

Line 132 Nucleotidic?
Line 307 systems
Line 323 There not they

Thank you, we have corrected these typos.

Reviewer #2:
Reviewer #2: Burgaya and coworkers aim to shed light into genome differences between E. coli commensal and BSI isolates. Authors take advantage of three French collections of isolates over a 17-year period. Importantly, the full sequence of the strains is available as well as some metadata. GWAS analysis and machine learning tools were used to conclude there is more genetic diversity in BSI isolates as well as some differences in the presence of so-called virulence genes, leading the authors to conclude that E. coli species evolved towards higher pathogenicity.

General comment.
Indeed, this is an interesting area of research, receiving increasing attention in a number of human pathogens. This is particularly relevant in the case of Enterobacteriacea that may reside in the gut in low numbers and that, upon dysbiosis, may cause invasive disease. In this regard, it is not apparent why authors expect to find differences between the gut-resident strains and the BSI ones, as the gut is the source of the later. This requires extensive clarification as the rationale of the study is in question. The only potential differences may relate to ARGs as they may evolve upon treatment of the BSI patient.

We agree it is critical to clarify even further the rationale behind our study.

We define *pathogenicity* as the capacity to cause infection, following the classical paper by Casadevall and Pirofski (1999) that we cited. Indeed, the gut bacteria are the source of BSI bacteria. However, **bacteria are variable in their pathogenicity and therefore the bacteria that are found in BSI are a selected sample of those found in the gut**.

More formally, our analyses are an instance of a **case-control study** where cases are the individuals with BSI, controls are the healthy individuals, and the exposure is *E. coli*. The exposure is variable because bacteria are genetically variable. If the exposure can be binarised as 0 or 1 (for example, bacteria without and with a given

virulence gene), we use the data to predict the outcome (no infection vs. infection) from the exposure with the logistic model:

outcome ~ exposure

The coefficient estimated from this logistic model can be exponentiated into the odds ratio quantifying the effect of the exposure (here, a given virulence gene).

Of course, the GWAS is more complex because it incorporates information on the whole genetic diversity of the species, but the basic rationale remains the same. **This is in fact the rationale behind all previous studies comparing bacteria in commensalism and in infection**, notably the GWAS studies cited in the Discussion.

We clarified the definition of pathogenicity and the rationale behind our analyses and previous work in the introduction lines 68-86.

> In this context, it is worth noting that a significant limitation/weakness of the present study is the fact that the same patient was not sampled for the isolation of the so-called commensals or the BSI isolate. The commensal collection represents the combination of 5 different collections over 30 years

No, this is incorrect, the five collections extend over 17 years not 30 years. The collection from 1980 was used to predict the level of pathogenicity further back in the past. The collections were explained in details in the original version of the manuscript. We further clarified the collection and bacterial isolation steps, which are similar across collections (line 535).

> ; authors already reported (PMID: 35862685) that the more
> recent isolates present increase frequency of so-called virulence associated genes than the isolates from the late nineteens.

The finding in the present paper relies on the pathogenicity score, a direct classifier of commensal vs. BSI strains. It is distinct and complementary to the previous findings which focused on virulence genes without direct assessment of their role in pathogenicity. The previous study only analyzed the commensal strains. The present study thus adds to the evidence that pathogenicity increases.

In addition, we have also made this analysis stronger as suggested by reviewer 3 by excluding the possibility that lower pathogenicity in 1980 could have been caused by collection/genome quality issues. We note the caveat that collections from 1980 present a signal of imperfect sample storage evidenced by mutations in the *rpoS* gene but this is unlikely to have affected pathogenicity (see above).

> The BSI collections are not contemporary to the commensal collection and for each of these collections different isolation protocols were followed (and they come from different countries).
> Therefore, in this work authors are not comparing contemporary collection of strains, and they are not from the same geographical region. One may argue that by probing

large collections this limitation will be mitigated; however it is not evident that the current study set up actually mitigates this significant weakness.

This is incorrect. All strains come from the same country and the vast majority from the Paris area. To avoid any misunderstanding, we clarified that “Brittany” is a region of France, not a distinct country. The same isolation protocols were used for commensals and for BSI as further clarified lines 545-551.

Another conceptual consideration is the way authors have clustered the strains, regarding the so-called commensals as less pathogenic/non-virulent. This reviewer strongly disagrees with this view. The gut environment is a hostile environment for Enterobacteriacea; therefore the survival in this harsh environment that includes a fierce competition with the resident gut microbiome cannot be regarded as less pathogenic as compare to the survival in the blood. As commented before, if the strains residing in the gut are those lnked to BSI it is difficult to understand why the pathogenicty/virulence potential is different in terms of genome content.

There is a misunderstanding of the concepts we use here. As explained above, pathogenicity is clearly defined, as the capacity of a strain to cause an infection, as in the classical paper from Casadevall & Pirofski (1999) which clarified the concepts of pathogenicity and virulence for microbes. Pathogenicity is inferred from a case-control design, as in many previous studies. Strains found in BSI are a selected sample of those found in the gut, because the strains from the gut have a variable propensity to cause an infection.

There is no denying that bacteria struggle for survival in the gut environment, may compete between themselves and other species, etc. We have proposed through several of our works that virulence can be considered as a by-product of commensalism.

Please note that this reviewer believes that there will differences in gene regulation between anatomical sites.

Excellent point, thank you for this remark. We now checked at genes upstream and downstream of associated unitigs in intergenic regions and found some hits. We have added a paragraph in the discussion section where we point to the important potential role of regulation in virulence with numerous relevant references.

Major comment.
1. The presence of one siderophores cannot be simply regarded as a virulence trait as different sets of strains may express different set(s) of siderophores. A better analysis of the data is needed to establish the repertoire of siderophores encoded by the panel of strains. If the repertoire is markedly different, authors need to validate experimentally whether this is translated into differences in fitness in iron restriction conditions. This reviewer anticipates the need to construct mutants as well as to generate chimeric

strains. 2. The same rationale applies to fimbriae. 3. This reviewer urges the authors to further characterize the role of some of factors identified (sopB, mltC and ompX) in blood survival and gut colonization.

We agree with the reviewer that the repertoire of VAGs should be considered in the study of virulence in *E. coli*, as virulence in this species could be the result of additive gene effects (doi: 10.1016/j.ijmm.2010.04.013 and Royer et al. Nat. Comms., in press). Therefore, we added a new analysis to evaluate the repertoire of adhesins and iron capture systems (the two most significant categories). As expected, we found a higher proportion of BSI than commensal strains carrying three to four systems of adhesins or iron capture systems. We also used a flexible lasso regression to investigate what combinations of virulence genes best predict pathogenicity. We found that the linear combination of *ecp*, *papGII*, or (with similar prediction ability) *sit*, *iuc* and HPI best predict pathogenicity. Note that we also tested models with both iron capture systems, adhesins, and interactions between them, within the lasso framework, but these models did not predict better the pathogenicity than the simpler models including only adhesins, or only iron capture systems.

Regarding the functional tests in animal models of gut colonization and sepsis, as explained in details above, such heavy experimental work is outside the scope of this paper. However, we list several evidence of the indirect link between the presence of these genes and pathogenicity in Table 1.

Reviewer 3

Reviewer #3: This work by Burgaya et al builds on three recent publications that examined correlations among commensal and bloodstream infection (BSI) E. coli isolate genomes and patient data. The current study combines previously analyzed datasets and some new methods, including a machine learning model, to link a number of bacterial sequence types, virulence and antibiotic resistance factors, and specific gene variants with the capacity of E. coli strains to cause BSI. Such studies could help explain why only some E. coli strains have the ability to cause disease, and could help improve diagnostics and vaccine development efforts.

Thank you for the overall positive assessment.

Most of the findings are expected based on previous work by this group and many others: specific Sequence Types and factors like iron acquisition factors are more likely associated with disease. This study does identify a number of additional novel genetic factors that are linked with BSI, but these are not validated as VAGs beyond establishing correlations from the literature.

This is the **first study comparing in a systematic manner and genome-wide a large collection of BSI and a large collection of commensals**. We disagree that most findings are expected. We particularly emphasize three novel findings that are enabled by whole genomes: (i) large heritability of pathogenicity: 70%, on the high end of the spectrum compared to other studies, and comparable to that for *S.*

pneumoniae for which a vaccine has successfully been deployed. We also note that heritability is contributed not only by ST structure, but by the whole phylogenomic diversity (ii) While AMR genes are over-represented in BSI strains, they are not when accounting for the population structure. (iii) Increased pathogenicity from 1980 to 2010. Only finding (iii) could reasonably have been expected based on our previous study (Marin et al. 2022), but we note that the evidence we provide here is quite different: in Marin et al. we find that some virulence genes increase in frequency; here we develop a pathogenicity score from comparison of commensals and BSI strains, and look at how this score evolves over time.

We have tried to clarify further in the introduction how our study compares to previous studies (in particular with respect to the design) and how it is novel: mainly by the size of the collection, the whole genomes, and the case-control design.

> The Discussion is well thought out and does a good job of pointing out many caveats of the study. However, some specific discussion of how this study differs from other published work in terms of the findings and approaches might also be helpful in explaining the importance of the findings. In the end, this paper does add to the field and helps define what it takes for E. coli strains to cause BSI, but the overall conclusions as presented are not too different from what the authors and others have already published.

We have tried to emphasize further in the discussion how our study was new, as highlighted above.

> Other specific issues.
> 1. One of the key findings noted in the Abstract is that the pathogenicity of commensal strains increased between 1980 and 2010. This is a remarkable finding if true, and one of the key things that sets this study apart from others that have correlated bacterial genomes with patient datasets. However, evidence that commensal strains are increasing in pathogenicity is sparse and not well substantiated. Multiple other variables might account for changes in the VAG makeup of commensals isolates in the 1980s versus strains collected later, including contamination and loss of VAGs associated with mobile elements due to collection issues

We agree one weakness is the fact that this trend is evidenced only in France. However, the increase is observed for both periods, 1980-2000 and for 2000-2010.

We have consolidated our analysis by verifying if the two factors mentioned by the reviewer, contamination and collection issues, could have caused such trends. We replied to these points above, and we re-iterate briefly that the collection and conservation schemes were similar for all collections in the 2000-2017 time period and that the samples were stored at -80°C with only one or two cultivation steps between the stool and the stored sample (now explained line 548 of the manuscript). We note that we did observe in the collection from 1980 a large number of samples with putatively deleterious mutations in *rpoS*, which is a known marker for poor sample storage. This is explained by the stab storage of these strains between 1980 and 2000 (the time at which they were frozen at -80°C). We also find a slight trend of

less genes in the past which could be explained by the loss of some genes. However, such a slight trend could not have modified our prediction of pathogenicity in 1980. All in all, we have therefore carefully reworded our conclusions and noted how the predicted increase, especially between 2000 and 2010 (a period unaffected by storage issues), is worth noting. This will stimulate the community to generate time-resolved datasets to attempt to replicate these tentative findings.

(e.g. were isolates frozen immediately or left on plates or in broth for prolonged periods, which could lead to loss of genes?).

This point is now clarified in the material and methods section. The strains are sub-cultured once or twice in rich medium, then immediately stored with glycerol at -80°C, with the exception of the collection from 1980 which was stored in stabs between 1980 and 2000.

2. Why was 2010 chosen as the cut-off data as a covariate in the study?

We chose the cut-off of 2010 because it is the median date represented in our dataset.

3. The paper uses a lot of jargon, especially with statistical and modeling approaches, that could be better explained to make the study more accessible to a broader audience.

Thank you for this comment. We checked and explained the technical statistical terms, including ‘unitigs’, ‘alpha’, ‘elastic net regularization’, ‘lasso’, ‘cross-validation’.

4. The discussion points (lines 345 - 347) suggesting that resistance may be co-evolving with pathogenicity determinants associated with specific clonal backgrounds is interesting. Are VAGs and resistance genes more likely to be associated within mobiles elements (IS, phage, or plasmids)?

Thank you, we directly checked the location of hits to investigate a potential genetic link between AMR and VAG genes. We found that in the dataset presented in the manuscript, which is composed exclusively of draft genomes, we see no instance of VAG and AMR genes co-occurring on the same contig. We however observe VAGs encoded in the vicinity of other VAGs (median distance 4832 bp) and AMR genes close together (median distance 1388 bp). We cannot of course exclude that there are instances in our genome collection in which VAG and AMR genes are close to each other, but we can assume it will be a rare occurrence. Overall this observation strengthens our hypothesis that virulence and resistance to antimicrobials may be coevolving because of the higher antibiotic use in patients.

5. The determination that a BSI originated from the gut versus the urinary tract is presumably based on the presence or absence of a UTI in the patient prior to or during

the BSI. Some explanation in the text of how this is determined would help. There are caveats to making these determinations that should be acknowledged, which could skew interpretation of the data. (e.g. could bacteria causing asymptomatic UTI cause BSI, or a patient with gut problems could also have UTI that are not related to potential BSIs).

The criteria for the determination of the portal of entry are now given in the material and methods section line 567. A citation of a work that we did on the Colibafi dataset comparing blood and portal of entry strains is now provided (doi: 10.1016/j.ijmm.2013.07.002). Note that our results justify the importance of the information on the portal of entry, as heritability is larger within each portal of entry, and the sets of genetic variants identified are distinct.

6. The orientation of the axis labels in Fig 3 could be flipped so that they are easier to read.

Done.

7. Lines 102-104: "…protocols adapted to the sample origin." It is not clear what this means. It seems unlikely that this is also true for samples analyzed form the 1980s.

We meant that the protocols are distinct for strains collected from blood and stool samples. More details are now given on each protocol, starting at line 535 in the material and methods section.

8. Some key points in the introduction should be better referenced - e.g. lines 58-59 lacks a reference.

Done, we added a reference for the fact that BSI with digestive portal of entry were more severe than BSI with urinary tract portal of entry line 60 (doi: 10.1128/JCM.01902-10).

9. Lines 72 and 385: "This design is interesting because it blocks hosts factors." Not clear what this means - did the authors mean that the experimental setup does not require consideration of host factors?

We developed a new analysis to clarify the performance of two commonly used designs to infer the bacterial genetic determinants of pathogenicity: the case-control design (that we used here) and the “case-crossover” design. In the case-crossover design, also used in many previous studies, patients with BSI act as their own control, thanks to bacteria isolated from their stool sample. We conducted a simulation study to analyze when we expect either design to have better power to identify bacterial genetic variants affecting pathogenicity, and better accuracy to estimate the effect of the variant (Results, new Supplementary Figure 1). We find that indeed the case-crossover design is unaffected by confounding, but suffers from low power when the number of colonising strains is typically small.

10. The section on page 13 (lines 257-268) is difficult to follow. Is this focused on only VAGs associated with B2 isolates?

We rewrote the corresponding section for clarity (now lines 317-320). The section is focused on all VAGs identified in the GWAS. The comment about B2 was that we had identified the two genes *iucB* and *papG* as significantly over-represented in BSI, in the targeted approach focusing on phylogroup B2. We hope this is clearer now.
